# Experimental Evolution of Gene Expression and Plasticity in Alternative Selective Regimes

**DOI:** 10.1371/journal.pgen.1006336

**Published:** 2016-09-23

**Authors:** Yuheng Huang, Aneil F. Agrawal

**Affiliations:** 1 Department of Ecology and Evolutionary Biology, University of Toronto, Toronto, Ontario, Canada; 2 Laboratory of Genetics, University of Wisconsin-Madison, Madison, Wisconsin, United States of America; University of California Davis, UNITED STATES

## Abstract

Little is known of how gene expression and its plasticity evolves as populations adapt to different environmental regimes. Expression is expected to evolve adaptively in all populations but only those populations experiencing environmental heterogeneity are expected to show adaptive evolution of plasticity. We measured the transcriptome in a cadmium-enriched diet and a salt-enriched diet for experimental populations of *Drosophila melanogaster* that evolved for ~130 generations in one of four selective regimes: two constant regimes maintained in either cadmium or salt diets and two heterogeneous regimes that varied either temporally or spatially between the two diets. For populations evolving in constant regimes, we find a strong signature of counter-gradient evolution; the evolved expression differences between populations adapted to alternative diets is opposite to the plastic response of the ancestral population that is naïve to both diets. Based on expression patterns in the ancestral populations, we identify a set of genes for which we predict selection in heterogeneous regimes to result in increases in plasticity and we find the expected pattern. In contrast, a set of genes where we predicted reduced plasticity did not follow expectation. Nonetheless, both gene sets showed a pattern consistent with adaptive expression evolution in heterogeneous regimes, highlighting the difference between observing “optimal” plasticity and improvements in environment-specific expression. Looking across all genes, there is evidence in all regimes of differences in biased allele expression across environments (“allelic plasticity”) and this is more common among genes with plasticity in total expression.

## Author Summary

Different developmental environments change how genes are expressed and what phenotypes are produced. Here we examine how the responsiveness of gene expression to different environments (“expression plasticity”) evolves in populations adapted to constant environments or heterogeneous ones (temporal or spatial heterogeneity) using experimental populations of *D*. *melanogaster*. We find the plastic response of the ancestral population that is naïve to both environments is generally opposed by the evolved differences between populations adapted to alternative environments. Populations that live in heterogeneous environments show evidence of adaptive expression evolution in genes predicted to evolve changes in plasticity.

## Introduction

Phenotypic plasticity is the phenomenon of one genotype producing different phenotypes (e.g., physical or behavioral) when exposed to different environments. To produce different phenotypes from a single genotype, one of the essential intermediate steps is the induction of gene expression changes by the environment [1,2]. Studying expression plasticity can provide insights on how different phenotypes are generated by the interactions between genotype and the environment [3]. Moreover, transcriptomics allow us to examine plastic responses for a large set of traits (expression levels of many genes) that are relatively unbiased compared to traditional phenotypic traits with respect to preconceived notions of their importance or ease of measurement, though the link between expression traits and ecological importance is typically more tenuous than for traditional phenotypes.

Plasticity may be beneficial, allowing organisms developing in different environments to produce phenotypes better suited to those environments. On the other hand, a plastic response can be deleterious if it shifts the phenotype away from the optimum for the organism, perhaps reflecting the inability of the organism to buffer against the imposed environmental perturbation [4,5]. Finally, a plastic phenotypic change can be neutral (or nearly so), e.g., a by-product of physiological response to the environment that has little effect on fitness [6]. A variety of transcriptional changes may occur as a result of exposure to a novel environment. Multigenerational selection in the new environment may engender genetic responses that reinforce initially beneficial plastic changes (e.g., genetic assimilation [5]). Alternatively, long-term selection may result in genetic responses that oppose deleterious plastic responses that were deleterious, resulting in a pattern of “counter-gradient” variation [7].

Plasticity can only be shaped adaptively in populations that evolve in a variable environment. A naïve population first exposed to a variable environment may initially exhibit beneficial plasticity with respect to some expression traits but deleterious plasticity for others. Subsequent evolution in a variable environment is expected to reshape plastic responses. For most traits, including expression traits, selection does not act directly on plasticity itself but rather plasticity evolves as a by-product of adaptation of trait means to each encountered environment [6]. How plasticity evolves depends on how the phenotypes initially produced in a novel environment differ from the optimal phenotype in each environment. Both decreases and increases in phenotypic plasticity could contribute to adaptation to variable environments [8]. Further, alternative forms of heterogeneity (e.g., temporal vs. spatial) may select on plasticity differently [9,10].

Although expression plasticity could be beneficial [11,12] or deleterious [13] and genetic variation for plasticity has been found in different organisms [14–17], we still have little understanding of how expression plasticity evolves, in terms of the rate and the directions. Can plasticity evolve adaptively on short time scales? Yampolsky et al. [18] used microarrays to survey the transcriptome of *Drosophila* populations maintained in homogeneous environments (regular or ethanol medium) or spatially heterogeneous environments (mixed of two types of mediums) for more than 300 generations but found that the selective regime had limited effect on expression plasticity for the two mediums. They suggested that evolution of expression plasticity might require a longer timescale.

Here we examine expression plasticity in experimental *Drosophila melanogaster* populations that have evolved under constant conditions or with either spatial or temporal heterogeneity in larval diets. We have previously used these populations to examine how environmental heterogeneity affects inbreeding depression [19], genome-wide molecular diversity [20], quantitative genetic variation [21] and adaptive potential [22]. Here we use these populations, after ~130 generations of evolution, to study expression plasticity in larvae. We address three types of questions:

1. For populations adapted to different (but non-varying) environments, are expression differences related to allele frequency differences? Do genetic differences in expression between divergently evolved populations reinforce or oppose the plastic responses of the naïve ancestor?
2. Is there evidence of either adaptive increases or adaptive decreases in plasticity in populations that evolved with environmental heterogeneity?
3. Does biased allele expression change across environments? Is this related to plasticity in expression levels?

## Results

**Fig 1:**
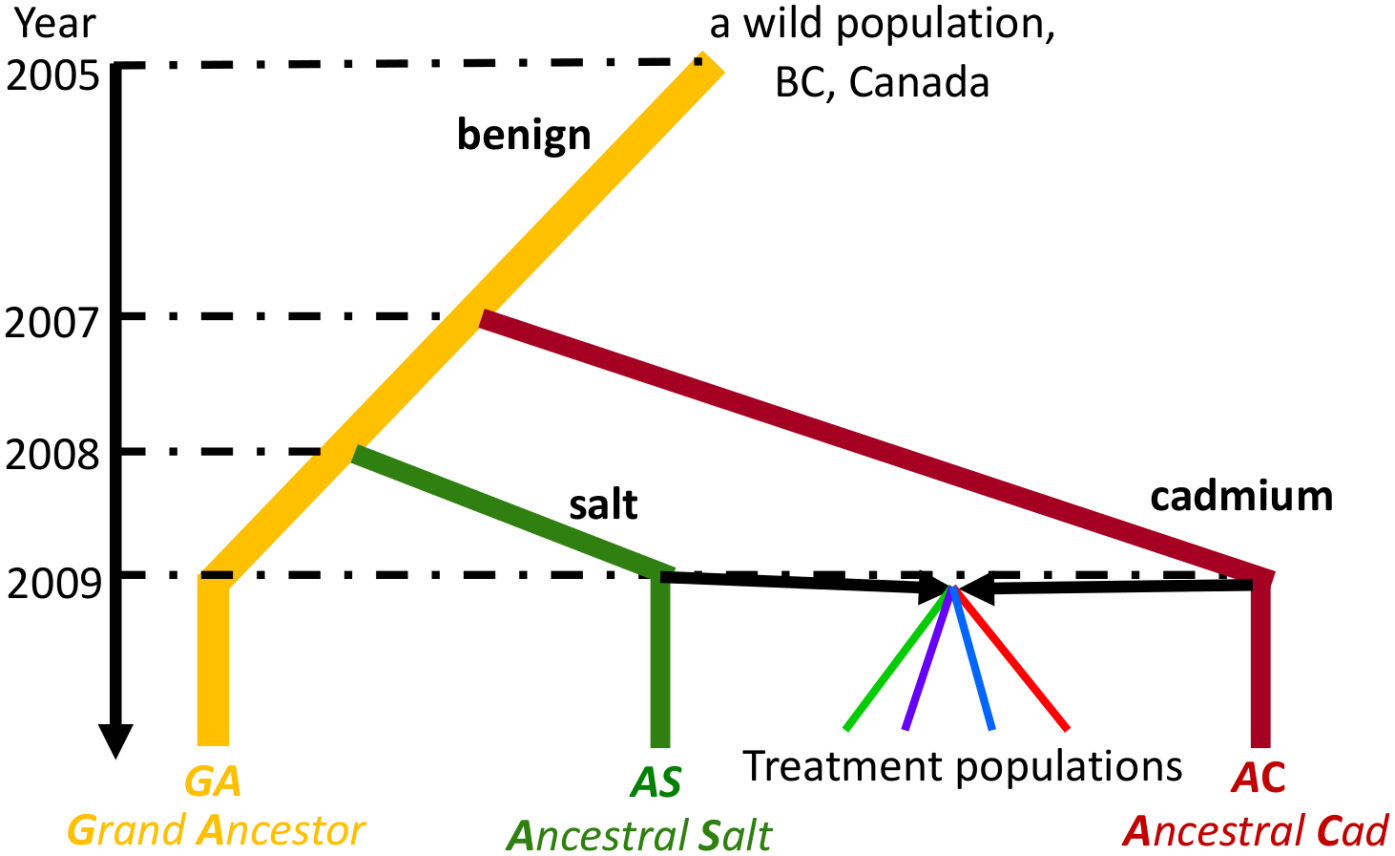
Selection history of the experimental populations. The *Grand Ancestor* population (*GA*) was established from wild collected flies and maintained in benign laboratory conditions (cornmeal diet). It was used to initiate populations maintained on cadmium-enriched diet (*Ancestral Cadmium* [*AC*]) or salt-enriched diet (*Ancestral Salt* [*AS*]). The treatment populations were produced by crossing two ancestral population *AC* and *AS*, and the F1 offspring were randomly divided among four selective regimes (*Cad*, *Salt*, *Temp*, and *Spatial*). There are five replicate populations of each of the four regimes (not illustrated).

The results reported here make use of a set of experimental fly populations, whose history is illustrated in Fig 1 and has been described in previous publications [19,20]. Briefly, the original field-collected population was maintained in a large lab population on standard cornmeal food (*Grand Ancestor*). From that population, two other large populations were established, one maintained on a salt-enriched diet (*Ancestral Salt* [*AS*]) and one on a cadmium-enriched (*Ancestral Cadmium* [*AC*]), and each of these adapted to its respective diet. A cross was made between *AS* and *AC* to create 20 smaller populations; these were divided among four selective regimes: *Cad*, *Salt*, *Temp*, and *Spatial* (five replicate populations per regime). In the two homogeneous selection regimes (*Cad* and *Salt*), larvae were reared on the appropriate regime-specific diet every generation. In the *Temp* treatment, populations were reared in alternating generations on salt- or cadmium-enriched medium. In the *Spatial* treatment, each generation half of the population was reared in one medium and half in the other, and the parents were mixed. Approximately 130 generations after the creation of these selective regimes, RNAseq data was obtained from each population, including the three ancestral populations. For each population, two RNAseq samples were obtained from very young larvae that had been exposed for 12 hours to either the cadmium- or salt-enriched diet (one sample of each).

### Differential gene expression between constant regimes

We first examine expression differences between populations that evolved under alternative constant conditions by contrasting the samples from the cadmium-selected (*Cad*) and salt-selected (*Salt*) populations. 546 genes show a “selection history” effect between *Cad* and *Salt* populations (FDR(*q*) < 0.1; we use a liberal *q*-value because we are interested in the properties of the genes in the list—tested in downstream analyses—rather than the genes themselves). A significant “selective history” effect can be loosely interpreted as an evolved difference in the level of expression averaged across diets. Previously, we examined allele frequency divergence between the *Cad* and *Salt* populations [20]. Combining those results with expression divergence, we tested whether the set of genes with significantly divergent SNP frequencies between the cadmium-selected and the salt-selected populations are enriched for genes showing divergent expression between *Cad* and *Salt*. We separated the genes based on whether the SNPs are located in exon, intron or intergenic regions. There is significant enrichment for differential expression for genes with differentiated SNPs located within 1kb outside the genic regions (*χ*^2^ = 8.0, df = 1, *p* = 0.0047 for genes with SNPs 1kb around the gene), but not for genes with SNPs located in exons (*χ*^2^ = 2.3, df = 1, *p* = 0.12) or introns (*χ*^2^ = 1.7, df = 1, *p* = 0.19). These results suggest that cis-acting factors contribute to the evolved divergence in expression (but these results do not exclude an important role for trans-acting factors as well). This is consistent with previous studies showing cis-regulatory variants are important for expression divergence between populations or species in *Drosophila* [23–25].

Next, we examine whether the direction of expression changes in the ancestral population when exposed to cadmium- versus salt-enriched food match evolved expression differences between cadmium- and salt-selected populations (i.e., do genes that are up-regulated by cadmium exposure relative to salt exposure in the ancestor evolve higher or lower expression in cadmium-selected populations than salt-selected populations?). Using the *Grand Ancestor* (*GA*) population, which is naïve to both diets, we identify 905 genes that have log_2_ fold change (log_2_FC) in expression between cadmium and salt diets greater than 0.4. (This plasticity reflects a change in expression then the ancestor is reared on cadmium versus salt; it is not a contrast with expression on benign cornmeal.) Using samples from the five *Cad* and the five *Salt* populations, there are 546 genes showing a “selection history” effect with *q* < 0.1. (The “selection history” history reflects differentiation between *Cad* and *Salt* regimes from one another, not necessarily differentiation from the *Grand Ancestor*.) A hundred and eight genes overlap between the two sets of genes (gene names are listed in S7 Table). The functional categories “Membrane” and “Transmembrane” are significantly enriched among these 108 genes (~40 of the 108 genes are related to membranes).

**Fig 2:**
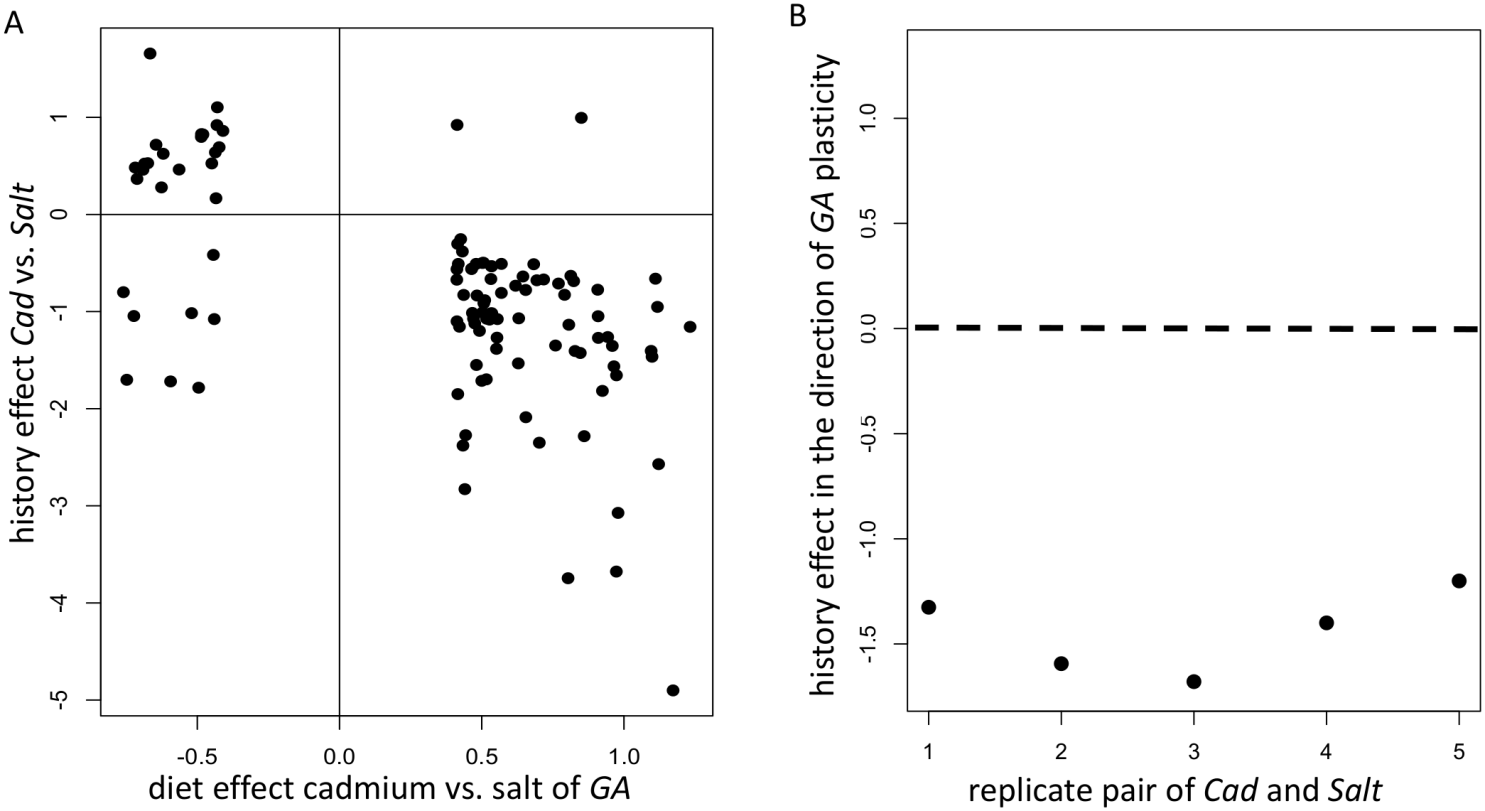
Plastic changes in the naïve ancestor (*GA*) compared to evolved differences in adapted populations. (A) The log2 fold change (log_2_FC) between diets in *GA* vs. the log_2_FC between regimes *Cad* and *Salt* from a statistical model using both diets. (B) The average expression difference between each *Cad* and *Salt* paired populations polarized in the direction of ancestral plasticity across the 108 genes. The expression difference is negative for all five pairs and the average across them is significantly below zero (*t* = -16.5, *p* = 8.0e-05) indicating that evolved differences between *Cad* and *Salt* is in the opposite direction to plasticity in the ancestor.

For 91% of these genes, the evolved divergence is in the opposite direction to the naïve plastic response (98/108; significantly different than 50%: *χ*^2^ = 71.7, df = 1, *p* < 2.2e-16; Fig 2A), meaning, for example, that a gene which is up-regulated when the ancestor is reared in cadmium (relative to salt) has evolved lower average expression in the *Cad* than in the *Salt* populations. Using *Cad/Salt* population pairs (rather than genes) as the unit of replication confirms this result where evolved responses oppose rather than reinforce plastic ones (Fig 2B). This type of “counter-gradient” response [7] is emerging as a major theme in evolutionary expression studies [26–29].

There are at least two reasons that a countergradient pattern could occur. If selection favours the same expression level (or phenotype) across environments but one environment induces a change in the phenotype, then opposing genetic changes are expected to evolve. Heuristically, genetic and environmental effects of opposite sign combine additively to yield little net change in phenotype/expression when genotypes are assayed in their adaptive environment. This type of explanation has been used for the countergradient pattern observed for the rate of tadpole development along an altitudinal gradient [30,31]. A different reason for the appearance of a countergradient pattern is related to the stress experienced by a population exposed to a novel environment. Abnormal expression responses occur because of the direct perturbation by the novel environment or reflect the stress response (or its cascading effects) employed to cope with environmental perturbation. Adaptation may render the environment benign (for example, via the evolution of efficient cadmium detoxifaction) such that there is no longer a direct perturbation or a stress response. In this case, genetic and environmental effects are not additive as each population is affected differently by the environment.

A closer inspection of our data indicates that the observed counter-gradient response does not appear to involve a genetic effect acting in opposition to but additively with the diet effect; rather *Salt* and *Cad* regimes are affected differently by diet (S1 Fig). In this gene set, a large proportion of genes is up-regulated in the naïve ancestor (*GA*) when it is reared in cadmium compared to when it is reared in salt; a similar pattern of plasticity is observed in *Salt* populations. In contrast, this plasticity has largely been evolutionarily lost in *Cad* populations such that there is little up-regulation of these genes in cadmium. A likely scenario is that, after *Cad* populations adapt reasonably well to cadmium, they no longer show the perturbed response of a population naïve to cadmium.

### Differential gene expression among experimental regimes

**Fig 3:**
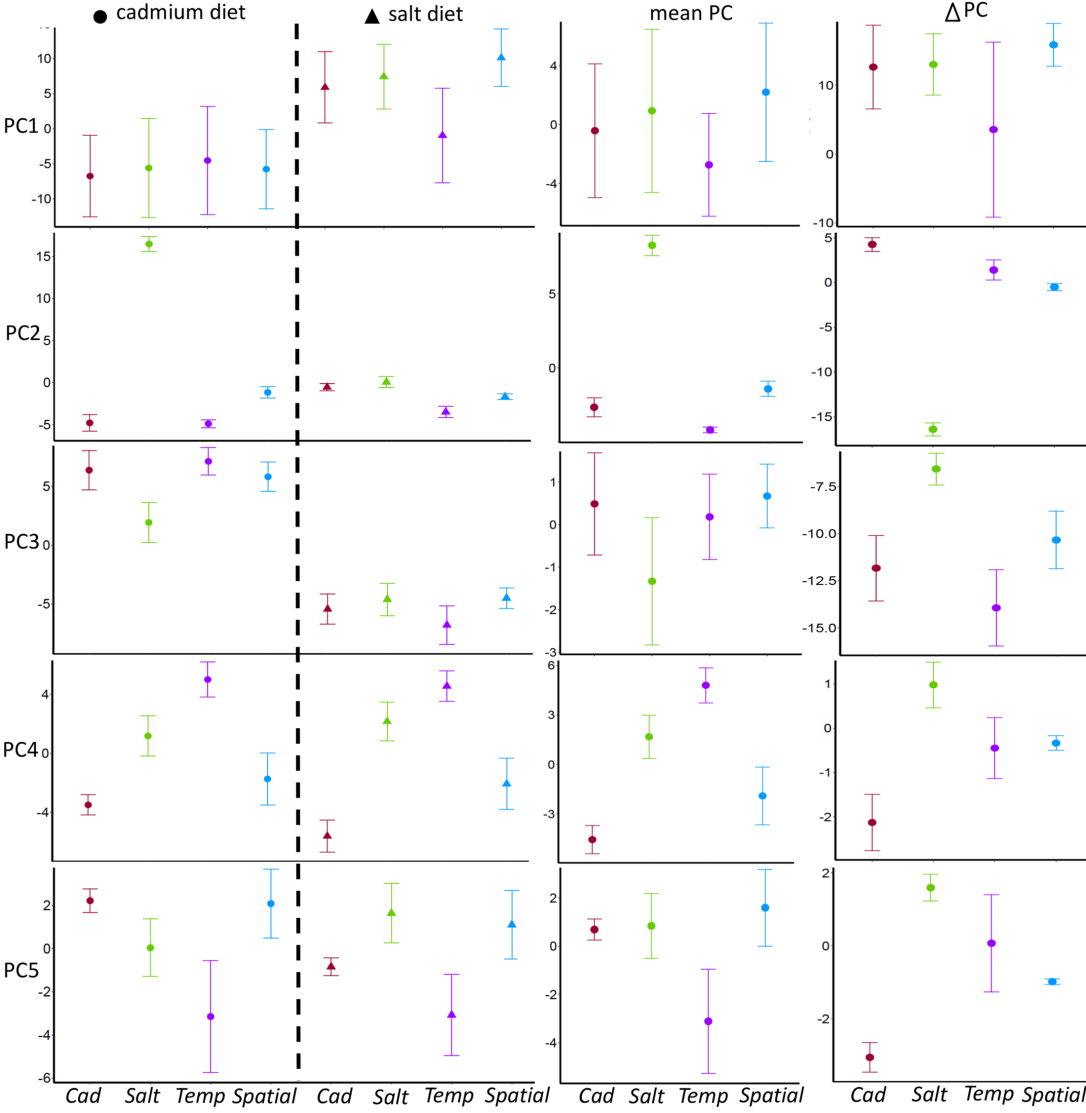
The first five principle component values for the experimental regimes. Principle components were determined using only the data from the experimental replicate populations (not the ancestors). Regime means (over five replicate populations) are shown for each diet (first two columns), as averages across diets (third column), and as the difference between diets (fourth column; value in salt minus value in cadmium). Error bars represent +/- one standard error among the five replicates per regime. The percentages of the total variance explained by PC1-5 are 41%, 10%, 9%, 4%, and 3%, respectively. PC1 partially reflects variation between diets (values in salt being larger than values in cadmium for most populations, i.e., ΔPC1 > 0). PC2 distinguishes *Salt* from the other regimes based on its strongly aberrant expression in cadmium. The remaining PCs show more subtle forms of diet-×-regime interaction. S1 Table highlights dimensions of difference for between pairs of regimes.

In addition to the two homogeneous selective regimes (*Cad* and *Salt*), this experiment involved two regimes with heterogeneous selection (*Temp* and *Spatial*). To visualize the expression divergence among experimental samples from all regimes, we plot the first five principle components of expression (Fig 3). The most striking pattern occurs with respect to PC2. *Salt* populations reared in cadmium have a very different value for PC2 than all other treatment/diet combinations, reinforcing the idea that cadmium perturbs the expression profile of populations without an evolutionary history of cadmium exposure. From further inspection of Fig 3, various effects of diet, regime, and their interaction are apparent; there are dimensions in which each regime differs from others either with respect to average expression across diets or plasticity between diets (S1 Table). A functional category enrichment analysis for the genes that strongly load on to each PC dimension is shown in S2 Table. A summary of gene-level pairwise comparisons among all four selective treatments is given in the Supporting Information and S3 Table.

A transcriptome-wide view of plasticity is given by the last column of Fig 3 but this perspective does not attempt to distinguish between genes showing plastic responses that are beneficial, deleterious, or selectively neutral. Though plasticity is expected to be more adaptive in the heterogeneous regimes than homogeneous regimes, we cannot test this prediction by looking at the transcriptome as a single unit. Rather, we need to first identify genes on which we expect selection to result in (i) increased or (ii) decreased plasticity. To do so, we leverage the history of our experimental treatments. The focal populations in all four selective regimes were originally created by crossing two diet-adapted populations, *Ancestral Cadmium* (*AC*) and *Ancestral Salt* (*AS*), followed by ~130 generations of selection within each of the four treatments. Using the expression patterns of the two diet-adapted ancestral populations (*AC* and *AS*), we screen for genes where we predict plasticity to evolve under heterogeneous environments and then examine levels of plasticity in these genes in our four experimental treatments.

### Plasticity evolution of potential targets for *increased* plasticity

If optimal expression differs across environments, then, in the absence of constraints, we would expect populations evolving in heterogeneous environments to evolve adaptive plasticity as a by-product of selection to produce different expression patterns in each environment [6]. Though we cannot know the “optimal” expression level, we can use expression of each diet-adapted ancestor assayed in its respective diet as a first approximation of the optimum. To identify potential targets for the evolution of increased plasticity in heterogeneous regimes, we used the data from the two diet-adapted ancestors (*AC* and *AS*). We screened for genes that met the two criteria (see Methods for further details). First, we required a reasonably large difference (|log_2_FC| > 0.4) in the “optimal” expression level for each diet (given by *AC* in cadmium and *AS* in salt). Second, to exclude genes that are initially highly plastic, we required relatively low levels of plasticity within each ancestor (the |log_2_FC| between diets for each of *AC* and *AS* is less than half the |log_2_FC| between *AC* and *AS* each in its own diet).

**Fig 4:**
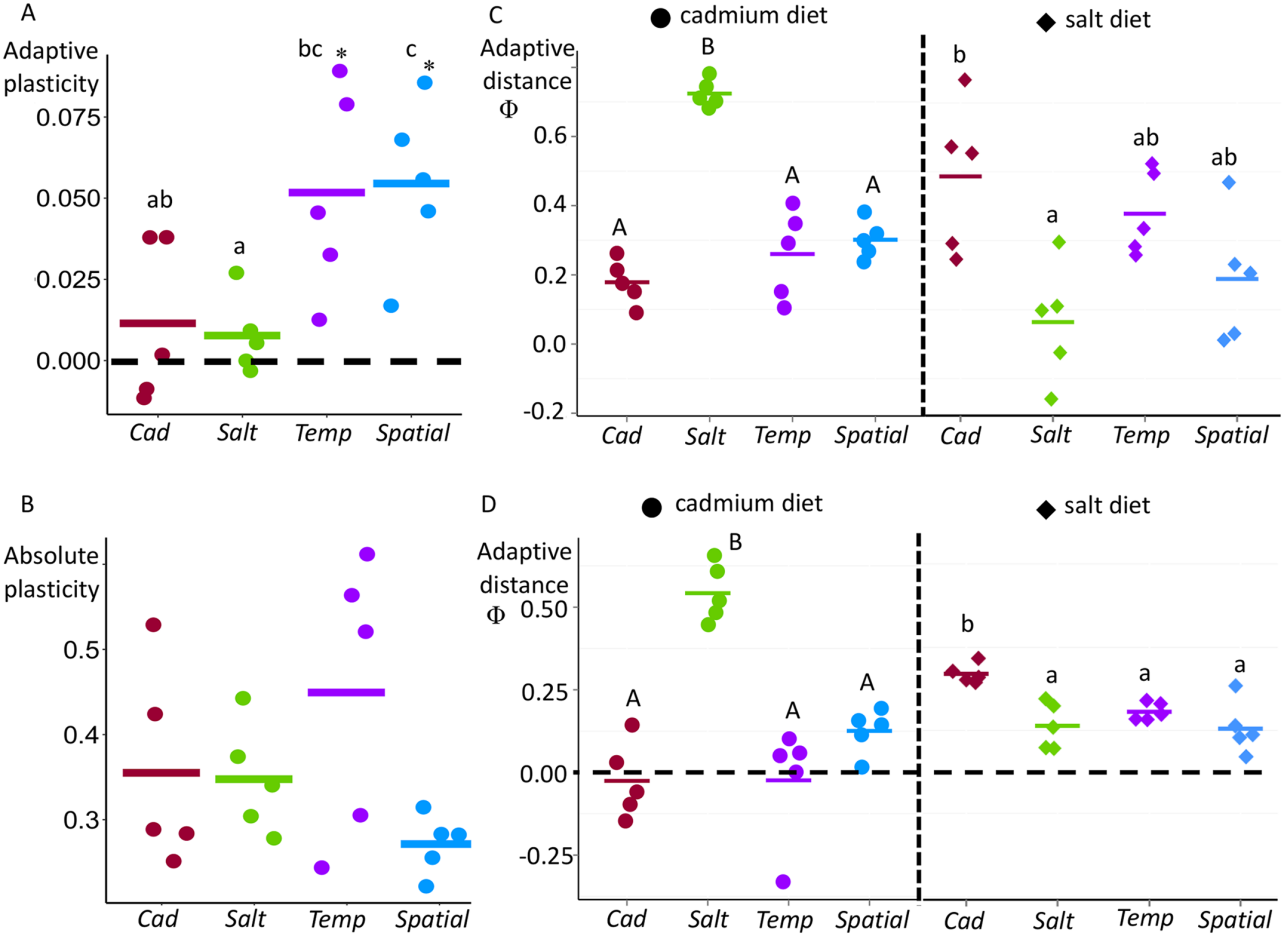
Plasticity and adaptive distance in expression for a set of genes expected to increase/reduce plasticity in heterogeneous regimes. (A) Log_2_FC across diets, polarized in the adaptive direction for each gene, and averaged across the set of genes that are expected to increase plasticity in heterogeneous regimes. There is significant variation among regimes; 49.5% of the total variance is caused by the variance between regimes (*F* = 6.2, df = 3, *p* = 0.009; letters denote statistically different groups). *Spatial* has significantly higher adaptive plasticity than *Cad* (*p*_adj_ = 0.047) and *Salt* (*p*_adj_ = 0.03). *Temp* has significantly higher adaptive plasticity than *Salt* (*p*_adj_ = 0.041) and the difference compared to *Cad* is marginally non-significant (*p*_adj_ = 0.065). The “*” indicates that the plasticity in the adaptive direction is significant from 0 based on a one-sample *t* test. (B) Average |log_2_FC| for genes expected to evolve reduced plasticity in heterogeneous regimes. The variation among regimes is not significant (*p* = 0.18), though the model attributes 30% of the variance to differences among regimes (C) Average adaptive distance $\bar{\Phi }$ for genes expected to increase plasticity. There is significant variation in $\bar{\Phi }$ across regimes in both diets (cadmium: *F* = 44.9, df = 3, *p* = 8.6e-07, 96% of the total variance is attributable to regime effects; salt: *F* = 6.3, df = 3, *p* = 0.008, 72% of the total variance is attributable to regime effects). In the cadmium diet, $\bar{\Phi }$ for *Salt* is significantly higher than for *Cad* (*p*_adj_ = 1e-06), *Temp* (*p*_adj_ = 5.8e-06) and *Spatial* (*p*_adj_ = 1.5e-05). In the salt diet, $\bar{\Phi }$ for *Cad* is significantly higher than for the *Salt* (*p*_adj_ = 0.009). (D) Average adaptive distance $\bar{\Phi }$. for genes expected to reduce plasticity. There is significant variation in average adaptive distance ($\bar{\Phi }$) among regimes in both diets (cadmium: *F* = 49.3, df = 3, *p* = 5.08e-07, 90% of the total variance is attributable to regime effects; salt: *F* = 9.8, df = 3, *p* = 0.0015, 81% of the total variance is attributable to regime effects). In the cadmium diet, $\bar{\Phi }$ for *Salt* is significantly higher than *Cad* (*p*_adj_ = 1.1e-06), *Temp* (*p*_adj_ = 1.1e-06) and *Spatial* (*p*_adj_ = 2.8e-05). In the salt diet, $\bar{\Phi }$ for *Cad* is significantly higher than for *Salt* (*p*_adj_ = 0.003), *Temp* (*p*_adj_ = 0.027) and *Spatial* (*p*_adj_ = 0.002).

This screen, based on the ancestral populations, identified 109 genes (S7 Table); no functional categories are significantly enriched for this set of genes. We now consider their plasticity in the four experimental treatments derived from these ancestors. For each gene in each population, we calculated the log_2_FC change across diets such that positive values indicate plasticity in the adaptive direction. For each population we averaged across the 109 genes to obtain a single measure of adaptive plasticity per population (i.e., “population”, not “gene”, is used as the unit of replication). The mean score for average adaptive plasticity is significantly greater than zero for both heterogeneous regimes (*t* = 3.6, *p* = 0.02 for *Temp*, *t* = 4.7, *p* = 0.009 for *Spatial*; Fig 4A). In contrast, the score is close to zero in both homogeneous treatments (*p* > 0.2 for each). A direct contrast of the heterogeneous versus the homogeneous treatments confirms the prediction that adaptive plasticity evolves to a greater extent in populations subject to variable environments (*χ*^2^ = 14.3, df = 3, *p* = 0.0025). The two alternative forms of heterogeneity (*Temp* and *Spatial*) appear to have very similar levels of adaptive plasticity.

### Plasticity evolution of potential targets for *reduced* plasticity

If optimal expression is similar in the two diets, then ideally there would be little or no plasticity. To identify potential targets for reduced plasticity in heterogeneous regimes, we again used the data from the diet-specific ancestors. We screened for genes meeting the following criteria (see Methods for details). First, we required that optimal expression was similar in the two diets. Second, to exclude genes that begin with little plasticity, we required that both ancestors (*AC* and *AS*) had a plastic response to the other diet that was large relative to the difference between the two optima. (Further, we required that both ancestors showed the same direction of plasticity between their adapted diet and their non-adapted diet; this requirement simplifies the interpretation of changes in plasticity.) This screen could include genes where selection always favours the same level of expression but that are misregulated under the stress of a novel environment. Alternatively, or in addition, this screen could include genes that are part of a stress response that is activated upon exposure to a novel diet but not when consuming a diet to which a population is adapted.

Using this ancestor-based screen, we obtained a set of 121 genes (S7 Table); no functional categories are significantly enriched for this set of genes. We now consider their plasticity in the four regimes. Because this gene set should ideally have little or no plasticity, we calculated the absolute value of expression change |log_2_FC| for each gene in each population, then averaged across the 121 genes to obtain a single value for each population. Though we expected to find lower values of plasticity in heterogeneous than homogeneous treatments, there was no evidence of this; *Spatial* had the lowest average plasticity and *Temp* had the highest average plasticity but there was no significant variation among treatments (Fig 4B). Based on these results it seems that expression has not evolved as expected in the heterogeneous regimes, especially not in the *Temp* treatment (but see below).

### Adaptive expression of potential targets of plasticity evolution

Plasticity measures the change in expression across diets but does not measure how adaptive expression is in either environment. To do the latter, we created a metric Φ to represent the relative distance to the optimum for expression in diet *d* of gene *i* of population *j*:

$$\Phi_{d,i,j}=\frac{E_{d,i,j}-O_{d,i}}{N_{d,i}-O_{d,i}}$$

where *O*_*d*,*i*_ is the expression for the sample representing the “*Optimal*” state for diet *d* (i.e., *AC* in cadmium diet or *AS* in salt diet) and *N*_*d*,*i*_ is the expression for the sample representing the “*Non-adapted*” state for diet *d* (i.e., *AS* in cadmium diet or *AC* in salt diet). When expression of a focal population is intermediate between values of the “*Optimal*” and “*Non-adapted*” states, the scaled distance to adaptive expression value is 0 ≤ Φ_*d*,*i*,*j*_ ≤ 1, with 0 meaning the expression in the focal population is at the “optimal” expression and 1 meaning the focal population is as poor as the non-adapted ancestor.

We first consider the set of 109 genes that we identified as potential targets to evolve increased (adaptive) plasticity. For each population, we calculated the average Φ_*d*_ over all the genes of interest for each diet separately. As expected for the constant regimes (*Cad* and *Salt*), $\bar{\Phi }$ values are close to 0 (optimal expression) in their respective adapted diets but the $\bar{\Phi }$ are far above 0 in the alternative diets (Fig 4C). Both *Temp* and *Spatial* regimes have $\bar{\Phi }$ values almost as low as the constant regime in its adapted diet, particularly in cadmium, providing evidence that heterogeneous populations are also relatively adapted to both diets for this set of genes.

We next consider the set of 121 genes that we identified as potential targets to evolve reduced plasticity. As expected for the constant regimes (*Cad* and *Salt*), $\bar{\Phi }$ is close to 0 (optimal expression) in their “native” diet but $\bar{\Phi }$ is far above 0 in the alternative diet, especially for the *Salt* regime in cadmium (Fig 4D). The expression for *Temp* and *Spatial* regimes are close to optimal expression ($\bar{\Phi }$ is close to 0) in both diets and significantly lower than the constant regime that is not adapted to that diet, suggesting that populations in heterogeneous populations are relatively well adapted to both diets with respect to expression for this set of genes.

### Differences in biased allele expression between diets

**Fig 5:**
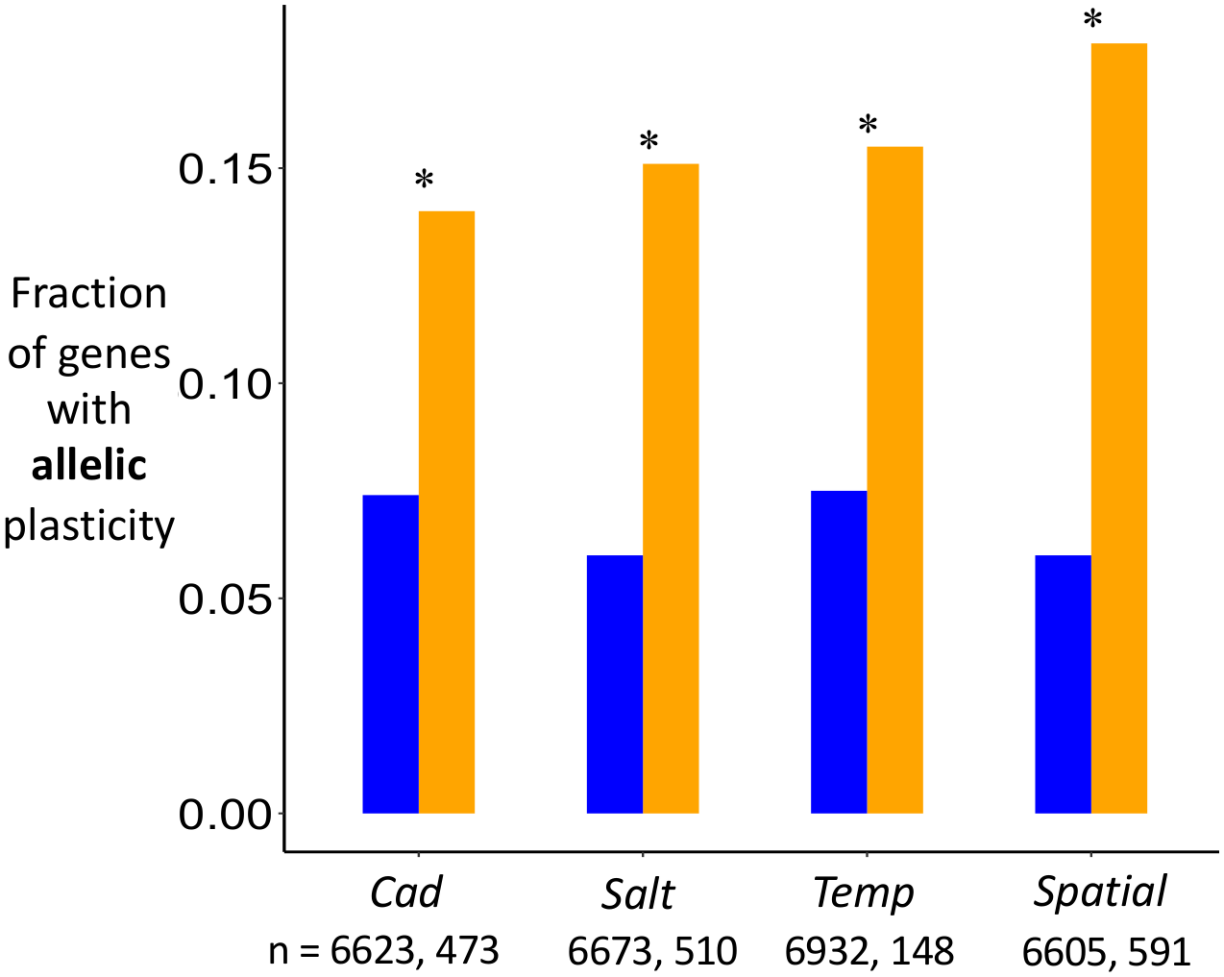
Enrichment of allelic plasticity among genes with abundance plasticity. For each regime, the fraction of genes with significant (*p* < 0.01) allelic plasticity among genes without (blue) or with (orange) significant (*p* < 0.05) abundance plasticity. Asterisks denote that permutation-based enrichment tests for each regime indicate allelic plasticity is significantly more common among genes with abundance plasticity in all regimes (*p* < 0.001 for all). The number of genes without (blue) or with (orange) significant (*p* < 0.05) abundance plasticity is given below each treatment label.

So far, we have focused on “abundance plasticity”, the difference in the total expression of a gene (summing across alleles) between diets. RNAseq provides us an opportunity to study another type of plasticity, “allelic plasticity”, which is the relative expression difference of two alleles for a polymorphic gene between diets (i.e., plasticity in allelic expression bias). This type of plasticity reflects variation in cis-regulatory elements whose effects are environmentally-dependent. Because each population is assayed in both environments, significant differences in SNP frequencies between environments in the RNAseq data reflects plasticity in allelic expression bias. In the context of pooled-seq data, allelic plasticity can be due to between-diet differences in expression between alternative homozygotes or because of between-diet differences in allele expression within heterozygotes. Alternatively, apparent allelic plasticity could be due to selection but there is little opportunity for this (see Methods). For each regime, we screened for polymorphic sites and then selected the most informative site within each gene. We detect evidence of allelic plasticity at numerous genes (~7% of genes tested); the average number of genes across the four regimes with significant (at *p* < 0.01) allelic plasticity is 516 whereas the average number of genes expected by chance based on a permutation analysis is 49. Moreover, allelic plasticity is approximately twice as common among those genes with significant abundance plasticity than those without (Fig 5). This pattern of enrichment would not be expected if a strong from of compensatory expression in which increased expression of one allele is balanced by reduced expression of the alternative allele in order to keep total expression reasonably constant. Rather, this enrichment likely exists because expression of one allele is substantially more sensitive to the environment but than the other, resulting in plasticity in total expression (abundance plasticity) as well as plasticity in the relative expression of the two alleles (allelic plasticity). These patterns are likely driven by variation in cis-regulatory elements. There is no indication that this enrichment varies among treatments.

We attempted to assess whether biased expression between diets is adaptive by examining the difference SNP frequencies in the RNAseq data between salt and cadmium assays for a given regime matched the direction of the difference in SNP frequencies from genomic data for salt- and cadmium-selected populations [20]. For the genes we identified as showing allelic plasticity (*p* < 0.01), the numbers showing significant allele frequency differentiation (*q* < 0.001) are: *Cad*, 204; *Salt*, 167; *Temp*, 196; *Spatial*, 177. However, for these genes we find no evidence, in any regime, that the allele favoured in a given environment is the more strongly expressed one. However, this method for detecting adaptive allelic expression bias is crude because it assumes that the favoured allele should be expressed more but in some cases reduced expression will be favoured.

## Discussion

This study demonstrates that alternative selective histories cause extensive divergence in gene expression and expression plasticity within 130 generations. We observe a striking pattern of countergradient variation with respect to expression divergence in a manner suggesting genetic responses evolve to restore an optimum perturbed by environmental effects. Though “countergradient” responses have been reported in several recent expression studies [26–29], not all studies use the term in exactly the same manner. Classic usage of the term implicitly assumes plasticity is constant (at least in sign) across populations [7] but in a recent study [28], and in our own, patterns of plasticity differ among populations. As suggested above, adaptation may often involve physiologically “managing” an environmental stimulus (e.g., cadmium detoxification) so that it no longer creates a cascade of expression perturbations. If so, we would not expect that the genes showing countergradient responses to be the direct targets of adaptive evolution. Ghalambor et al. [28] argued that genes that have their expression maladaptively perturbed by a novel environment would be under strong selection to evolve genetic changes in expression. From this perspective, one might expect many of the genes with countergradient responses to be the direct targets of adaptive evolution. Future studies attempting to resolve these issues should strive to understand mechanistically (i) why plasticity occurs, (ii) what genetic changes underlie adaptation, and (iii) how these are responsible for changes in transcription.

The expression perturbations experienced by a naïve population relative to an adapted one are not necessarily bad, and some may be beneficial in the short-term. Naïve populations may respond to novel environmental stimuli by altering gene expression in helpful but non-ideal ways that mitigate harmful environmental effects, e.g., beneficial stress responses that are no longer needed (and possibly harmful) after better mechanisms of coping with the environment have evolved. Such genes could contribute to the gene set used in our countergradient analysis as well as the gene set predicted to evolve reduced plasticity, though there is no obvious indication of this from GO analysis. Regardless, these expression changes reflect the lack of a good solution to the environment even if some expression changes are beneficial initially. Thus, inclusion of such genes does not interfere with the objective of either analysis, though this possibility should be considered in interpreting the results. Specifically, we cannot assume that plastic responses that occur a naïve population but not in an adapted one are all necessarily deleterious in the naïve population. Expression studies like our own cannot identify which specific plastic changes are beneficial or deleterious in populations with different genetic backgrounds. In principle, selection on gene expression within a population could be studied using the same framework used to study selection on traditional phenotypes [32] though in practice this would be very difficult given the high dimensionality.

Changes in transcriptome-wide expression plasticity can be difficult to interpret because selection that results in adaptive reaction norms will not occur in populations living in homogeneous environments. For populations experiencing heterogeneity, selection within each environment may ultimately result in increases or decreases in plasticity for different genes. Our approach has been to use the diet-adapted ancestors as a guide to optimal expression within each environment. Using this approach, we inferred the ideal level of plasticity in the absence of constraints and identified gene sets that we expected to evolve increased or reduced levels of plasticity. Focusing on these gene sets, we found that expression in heterogeneous regimes was more adaptive in each environment than that of the non-adapted homogeneous regime. For genes predicted to increase plasticity, we found, as expected, higher levels of adaptive plasticity in heterogeneous regimes than homogeneous regimes. However, we did not find a reduction in plasticity in heterogeneous regimes for genes predicted to reduce plasticity despite the evidence of adaptive levels of expression.

Two reasons may contribute to the seeming discrepancy with respect to this latter gene set for which we see evidence of adaptive expression yet not the expected reduction in plasticity in heterogeneous regimes. First, any measurement error in expression (in addition to true plasticity) will contribute to our estimates of |log_2_FC| because we are using the absolute value of the difference in observed expression values between diets. Though such error should not artificially create differences in |log_2_FC| among regimes, it may reduce our power to detect true variation among regimes. Second, the observed results could arise simply by evolution toward optimal expression proceeding faster in one environment than the other within heterogeneous regimes (S2 Fig). Consider a case where expression in both environments is initially higher than the optimum, which is the same in both environments. If expression levels evolve down toward the optimum in both environments but adaptation proceeds faster in one environment than the other, then this would result in increased plasticity in expression across environments despite improvement in both. The observation of adaptive expression without the expected reduction in plasticity serves as a reminder that selection does not truly act directly on plasticity for most types of traits [6]. If improvement is possible in only one environment, then this may result in increased plasticity (at least transiently) even if optimal expression is the same in both environments.

Because the averaging across environments works differently with temporal and spatial heterogeneity [33], plasticity may evolve differently with these alternatives forms of heterogeneity, though existing models make different predictions about the nature of this difference [9,10]. With respect to expression in the gene sets predicted to evolve increased and decreased plasticity, we see some hints of differentiation between the *Spatial* and *Temp* treatments (Fig 4) but these are not statistically significant. Given that there are SNP frequency differences between the *Spatial* and *Temp* treatments [20], a remaining challenge is to understand mechanistically how and evolutionarily why adaptation occurs differently with alternative forms of heterogeneity. When other studies investigate differences in plasticity in populations with spatial versus temporal heterogeneity, it will be possible to ask if there are general patterns in how plasticity evolves with these two common forms of heterogeneity.

A fundamental question of expression evolution is the relative importance of cis and trans effects. Previous work has established several lines of evidence that cis effects are of considerable importance [23–25]. Two of our results add to this. First, we observed that genes differentially expressed between *Cad* and *Salt* regimes where enriched having significantly differentiated SNP frequencies located in nearby intergenic regions. Second, we found evidence of extensive diet-dependent differences in allelic bias and that this allelic plasticity is associated with abundance plasticity. The simplest explanation for these observations is environmentally-sensitive cis-acting factors. This observation, along with other recent studies [34,35], raises the possibility that a substantial fraction of the genetic variation for expression may be manifest under particular environments (i.e., a large G×E component).

Our view of expression plasticity in this study is limited in several respects. We have measured expression at only a single developmental stage (very young larvae) and patterns may differ at other stages. For example, we see evidence that expression of *Salt* larvae is strongly perturbed in cadmium but comparatively little expression perturbation of *Cad* larvae in salt. Because we know that egg to adult survivorship is low for both situations [21,22], more severe expression perturbations are expected for *Cad* larvae in salt but these may not become apparent until later in development. Second, our analysis, like most expression studies, is biased towards detecting expression differences for genes that are reasonably highly expressed. Third, the pattern of plasticity evolves over time and may not yet have reached its equilibrium so measuring plasticity at multiple time points for evolving populations would be informative; this study represents only a single snapshot of expression evolution. Despite these limitations, various patterns are apparent and we have no *a priori* reason to believe these are misrepresentative, though other patterns might emerge with other types of expression data.

While we have studied expression and its plasticity in well-controlled experimental populations, comparing expression plasticity for populations living in different natural habitats should generate insights into how plasticity facilitates adaptation on long timescales [26,27]. What aspects of the regulatory networks mediating plasticity evolve differently in short versus long evolutionary timescales? Combining different approaches from laboratory experiments to field studies should yield a more comprehensive understanding of the selective forces and constraints on the evolution of plasticity.

## Materials and Methods

### History of selection populations

A full description of the selective history of these populations can be found in Huang et al. 2014 [20]. Briefly a population collected from the wild was maintained in standard benign conditions (approximately 2000–4000 adults), referred to here as the “*Grand Ancestor*” (*GA*). Two subsets of flies from the *GA* population were used to initiate a population maintained in a cadmium-enriched medium and a population maintained in a salt-enriched medium, each with population size ~1000, referred to as the “*Ancestral Cadmium*” (*AC*) population and the “*Ancestral Salt*” (*AS*) population, respectively. 448 males and 448 virgin females were collected from each the *AC* and *AS* populations and crossed with flies from the other population. The offspring from the next generation were used to found 20 populations (each with 448 adults) that were distributed evenly among four regimes: (i) constant cadmium-enriched (CdCl_2_) medium (“*Cad”*), (ii) constant salt-enriched (NaCl) medium (“*Salt”*), (iii) alternating each generation between salt- and cadmium-enriched media (“*Temp”*), and (iv) half the rearing vials containing cadmium-enriched medium and the other half containing salt-enriched medium (“*Spatial”*). For the *Spatial* regime, an equal number of adult flies produced from each type of medium were mixed to produce offspring for the next generation (i.e., a “soft” selection regime *sensu* [36]). The *Cad* and *Salt* regimes show the expected patterns of local adaptation based on the different fitness assays measured at ~50 and ~130 generations, i.e., populations from each regime perform better in their own diet than in the alternative [21,22]. The *Temp* and *Spatial* regimes have intermediate fitness between the two constant regimes in both diets. This suggests that the heterogeneously selected populations are relatively well adapted to both diets, though less adapted than the homogeneously selected populations in their own selective diets.

### Sample preparation and RNA-sequencing

Starting at ~127 generations of experimental evolution, samples were prepared in five blocks. Each block used one replicate population from each of the four regimes and assayed in both diets, resulting in eight samples (perfectly balanced) per block. Block 1 was performed at generation 127; Blocks 2 and 3 were performed at generation 128; Blocks 4 and 5 were performed at generation 129. Samples from all three ancestral populations were prepared at generation 129, regarded as Block 6. One generation before the collection, each replicate experimental population was reared in regular cornmeal diet to control for the maternal environment. In next generation, the emerged adult flies (Day 12) of each population mated for 1.5 days and then laid eggs on either salt or cadmium treatment diet. Before the actual egg-laying, pre-lay plates were supplied for two hours to provide ample opportunity for females to dump any stored eggs and ensure synchronization of the developmental stage of subsequently laid eggs. Laying plates were then supplied for two hours before the adults were removed. After ~ 20 hours (at 25°C), any hatched larvae were removed and discarded. Newly hatched larvae were transferred to the same selective diet within a one-hour window. After 12 hours, 80 larvae were collected per sample in 1.5 ml tubes with PBS solution (i.e., all larvae were 12–13 hours old). The PBS solution was removed after centrifuging at 9500 rmp for 1 min. The samples were immediately frozen in dry ice and store in -80°C prior to RNA extraction. Total RNA was extracted using the NucleoSpin RNA Kit (MACHEREY-NAGEL). Strand-specific single-end libraries were prepared and sequenced in six lanes of HiSeq2000 (The McGill University and Génome Québec Innovation Centre). All eight samples from the same block were sequenced in the same lane except the sample of replicate population 2 of *Temp* in cadmium diet treatment because of an error at the sequencing centre. The six samples from the ancestral populations were sequenced in one lane.

### Read mapping and down-sampling

The single-end reads were mapped to the *D*. *melanogaster* transcriptome and genome (*FlyBase* release version 5.41) using Tophat2 with library-type as fr-firststrand [37]. Only the alignment with the highest alignment score was used. If multiple alignments with the same score existed, one alignment of them was randomly retained. The aligned reads were sorted and viewed using samtools v. 0.1.16 [38] and then assigned to features of the transcriptome using HTSeq with default settings [39]. Because differences in coverage among treatments can result in different statistical power, we performed down-sampling of the mapped reads to obtain equivalent level of coverage across diet treatments and selective regimes. We first ranked the eight samples per experimental replicate block by read number. For each block *i* (*i ϵ* {1–5}), we found the minimum coverage across the eight samples, *n*_*i*_. We sampled without replacement *n*_*i*_ reads from the *i*^*th*^ block for each sample. As a result, the numbers of mapped reads remaining per sample within each block were as follows: block 1, 21707402; block 2, 23714035; block 3, 22732039; block 4, 23378572 and block 5, 20909145. The numbers of useful reads for the six samples from the ancestral populations (*Grand Ancestor* (*GA)*, *Ancestral Cad* (*AC)* and *Ancestral Salt* (*AS)* in both diets) were in the range of 30.7 to 33.5 million.

### Differential gene expression analysis

The gene expression counts were analyzed by the *DESeq2* package [40] of the BioConductor suite [41] with empirical Bayes estimation. The expression counts for each gene were normalized as a quantity proportional to the concentration of cDNA from the gene in each sample and transformed to log_2_ scale [40]. To examine divergence between each pair of selective regimes, the transformed expression value for each gene of the 20 samples (two regimes each with five replicate populations in two diets) was analyzed by a generalized linear model with a logarithmic link: Expression ~ diet + selective history + diet × selective history + block. The selective history effect represents the difference between regimes in expression averaged across the diets. To highlight whether expression differs between regimes in one diet more than the other, we also examined expression separately for each diet: Expression ~ selective history + block. To examine levels of parallel plasticity among five replicates within each regime, we examined each regime alone (10 samples): Expression ~ diet + block. The Benjamini-Hochberg procedure was used to control the false discovery rate (FDR. i.e., *q*-value; [42]) in R (version 3.2.0, R-Development-Core-Team 2015). Gene expression changes between diets or regimes were calculated as log_2_ fold changes (log_2_FC) between two tested groups.

To examine whether the initial plasticity in the *GA* population tends to be reinforced or opposed during adaptive differentiation, we screened for genes that have log_2_FC between the two diets greater than 0.4 in *GA* and show a strong “selective history” effect (*q* < 0.1) in linear model of expression comparing *Cad* and *Salt* populations. To compare plasticity of the *GA* to evolved differences between *Cad* and *Salt*, we used the log_2_FC between diets for the *GA* and then calculated the log_2_ fold change between the replicate *Cad* population and the replicate *Salt* population for gene *i* in block *j* as

$${\text{log}}_{\text{2}}{\text{FC}}_{i,j}=\;½Z\left({\text{log}}_{\text{2}}\left(E_{cadmium,i,Cad\_j}\right){\;+\text{ log}}_{\text{2}}\left(E_{salt,i,Cad\_j}\right){-\text{log}}_{\text{2}}\left(E_{cadmium,i,Salt\_j}\right){-\text{log}}_{\text{2}}\left(E_{salt,i,Salt\_j}\right)\right)$$

where *E*_*d*,*i*,*j*_ is the normalized expression in diet *d* for gene *i* in population *j* (number of expression counts divided by the total number of counts of the sample). *Z* serves as an indicator of whether plasticity in *GA* is in the same or opposite direction of adaptive divergence between *Cad* and *Salt*: *Z* = 1 if expression was up-regulated in cadmium for *GA* and *Z* = -1 if expression was down-regulated in cadmium for *GA*. We averaged the expression changes across the screened genes for each replicate pair (i.e., block).

### Principle component analysis

To visually assess the overall patterns of variation in the transcriptome among samples, we first performed principle component analysis for all samples, including the ancestral populations, using *DESeq2*. The *DESeq* dataset object was constructed from the matrix of the count data and the sample information table, with design format as ~ regime + diet. After *regularized-logarithm transformation* (*rlog*), the top 1000 genes with highest variance across samples at the transformed scale were used for principle component analysis (PCA). The principal component value for each sample was obtained by the function *plotPCA*. The values for all samples with respect to the first and second principal components are plotted in S3 Fig. The samples from ancestral populations are somewhat distinct from the experimental population samples along the PC1 axis. The separation between samples from ancestors and experimental populations may be due to subtle life history differences because the ancestral populations are maintained slightly differently (in terms of density and other maintenance procedures) or because the ancestral populations were collected for RNAseq in a different week (i.e., block effect). To qualitatively assess whether block effects tend to be large, we repeated the same PC analysis without the ancestors, with the design format changed to ~ regime + block. From visual inspection, there is no indication of strong block effects among the experimental populations, either in the PCA above or in a PCA based on only the experimental populations, i.e., excluding the ancestors (S4 Fig). This PCA (without the ancestors) is the one represented in Fig 3. To further explore the functionality of different PC axes, we extracted the loading value for each of the 1000 genes on different PC axes using *prcomp* function. Using the R package “gage”, we tested, for each PC, whether different GO Ontology categories were significantly associated with either positive or negative loadings on that PC.

### Examination of genes predicted to evolve increased or decreased plasticity

To identify genes expected to evolve increased plasticity in heterogeneous regimes, we used a screen based on the *Ancestral Cadmium* (*AC*) and *Ancestral Salt* (*AS*) populations. We treated the samples of *Ancestral Cadmium* (*AC*) in the cadmium diet and *Ancestral Salt* (*AS*) in the salt diet as “*Optimal*” and samples of *Ancestral Cadmium* (*AC*) in the salt diet and *Ancestral Salt* (*AS*) in the cadmium diet as “*Non-adapted*”. We identified candidate genes that should be selected for increased plasticity in heterogeneous regimes by finding genes that meet the following criteria: (i) large expression differences between the two “*Optimal*” states (|log_2_FC| > 0.4); and (ii) low levels of plasticity relative to regime effects (the |log_2_FC| between diets for both *AC* and *AS* populations is less than half the |log_2_FC| between *AC* and *AS* within each diet). The cut-off values used represent a compromise between high stringency to obtain a set of genes with the desired properties and ensuring a reasonable number of genes (~100) pass the screen to allow for meaningful downstream analysis. For this set of genes, we calculate the relative adaptive plasticity (polarized plasticity) as:

$${\text{log}}_{\text{2}}{\text{FC}}_{i,j,p}\text{=}Y{*\text{ log}}_{\text{2}}{\text{FC}}_{i,j}$$

where *Y* = {-1, 1} is an indicator of whether the direction of plasticity matches the direction of difference between the two “*Optimal*” ancestral states. log_2_FC_*i*,*j*_ is the log2 fold change for gene *i* in population *j*, calculated by *DESeq2*. We calculated the average log_2_FC_*i*,*j*,*p*_ across the genes for each population. The paired comparisons of the average log_2_FC_*i*,*j*,*p*_ among selective regimes were based on ANOVA Tukey HSD tests. To test whether the heterogeneous populations differ from homogeneous populations in relative adaptive plasticity, we analyzed the average polarized plasticity among the gene set $\bar{log_{2}FC_{i,j,p}}$ using the lmer function in the lme4 package in R:

$$\bar{log_{2}FC_{i,j,p}}\;=\text{ regime }+\text{ selective history }+\text{ block}$$

where regime is homogeneous or heterogeneous; selective history (*Cad* or *Salt*, *Temp* or *Spatial*) is nested within the homogeneous (*Cad* and *Salt*) or heterogeneous regime (*Temp* and *Spatial*); block was treated as random effect. The regime effect was tested by comparing the full model with a model without the regime effect.

To identify genes expected to evolve reduced plasticity in heterogeneous regimes, we again treated the samples of *AC* in the cadmium diet and *AS* in the salt diet as “*Optimal*” and *AC* in the salt diet and *AS* in the cadmium diet as “*Non-adapted*”. We used two liberal criteria to screen for genes for hypothesis testing: (i) genes must be differentially expressed between the “*Optimal*” and “*Non-adapted*” states: |log_2_FC| > 0.3 and the difference between “*Optimal*” and “*Non-adapted*” must be in the same direction for both *AC* and *AS* (i.e., “*Optimal*” states both have higher or both have lower expression than the “*Non-adapted*” states); and (ii) differences in the adaptive state of the ancestors must be relatively low: the |log_2_FC| between “*Optimal*” states for *AC* vs. *AS* must be less than half as large as the |log_2_FC| for “*Optimal*” vs. “*Non-adapted*” for both *AC* and *AS*. For each gene passing the two criteria, we calculated the scaled absolute plasticity (|log_2_FC|) based on *DESeq2* and took the average across genes for each population. The comparisons among selective regimes were based on ANOVA Tukey HSD tests.

To further examine how selective history alters expression on genes of interest, we calculated the scaled distance to the “adaptive” optimum for expression in diet *d* of gene *i* of population *j* (Φ_*d*,*i*,*j*_):

$$\Phi_{d,i,j}=\frac{E_{d,i,j}-O_{d,i}}{N_{d,i}-O_{d,i}}$$

where *O*_*d*,*i*_ is the expression for the sample representing the “*Optimal*” state for diet *d* (*AC* in cadmium diet or *AS* in salt diet) and *N*_*d*,*i*_ is the expression for the sample representing the “*Non-adapted*” state for diet *d*. For each population, we calculated the average Φ_*d*,*i*,*j*_ over all the genes of interest for each diet separately. The average value of Φ_*d*,*i*,*j*_ across all genes in each gene set was calculated for each population. These average values were used in comparisons among regimes.

### GO enrichment test

Gene Ontology enrichment test was performed with the R package “gage” [43] with ranked based two-sample t-test. Different sets of genes were tested for functional enrichment: genes for principle component analysis, genes for differential expression analysis between diets/regimes. Selection of overrepresented GO terms among all the tested GO (only considering the terms that do not associate with the child terms) was based on FDR(*q*) < 0.05 and were reported on different directions separately (i.e., positive or negative loading values on each PC axis; up-regulated in one or the other diet/regime). For the genes involved in comparing initial plasticity in the *GA* and evolved divergence, we obtained the functionality information and tested for overrepresented GO terms using the Database for Annotation, Visualization and Integrated Discovery (DAVID) [44,45]. We performed the same GO analysis for the gene sets predicted to evolve increased or decreased plasticity.

### Allelic bias expression analysis

To identify genes with different levels of allele expression bias in different diets (“allelic plasticity”) for each regime, we assumed the allele frequencies are the same for the two samples in different diets from a replicate population. Therefore, the difference in the ratio of mRNA levels of two alleles between diets is due to expression changes but not DNA. There will be some difference due to sampling of larvae (“genetic drift”) but this should be minimal because 80 larvae where taken for each sample. More importantly, the direction of drift should not be the same across replicates (reducing the statistical power to detect allelic plasticity rather than creating false positives). It is possible that during the 12 hours of diet feeding, selection changed the allele frequency. However, the opportunity for selection seems very small as dead larvae were rarely observed and almost all the larvae on the medium were collected for RNA extraction. However, if different genotypes grow at different rates at different diets, the changes in the allelic expression ratio will be due to the changes of relative contribution of mRNA from different genotypes. Bearing this caveat in mind, we examined between-diet differences in allelic expression within each regime. (An additional analysis described below found no evidence of “allelic plasticity” being in the direction of the allele favoured in a given diet, providing further evidence that selection is not responsible for observed instances of allelic plasticity.) To test for allelic plasticity, we first used Popoolation2 [46] to obtain the counts for different nucleotides in each position of the genes. To control the statistical power of identifying allelic expression among diets, we down-sampled the nucleotide counts for each site to the minimum coverage of the sites in each block (eight samples). For all the samples from regime *i* in both diets, we screened for sites that (i) have average diversity 2*p*_*i*_*q*_*i*_ > 20% (where *p*_*i*_ is the average mRNA nucleotide count frequency across the 10 samples for regime *i*, *q*_*i*_ = 1 –*p*_*i*_), (ii) the total count for the site passes *n*_*i*_ > 100 (where *n*_*i*_ is the total count for that site among the 10 samples for regime *i*), and (iii) *n*_*i*_ is at least half of the total read count for that gene. We chose the most informative site within each gene for each regime (highest *n*_*i*_*p*_*i*_*q*_*i*_), to calculate its allelic expression. Significant allelic plasticity was identified by Cochran-Mantel-Haenszel (CMH) test in R (*p* < 0.01). Two samples in different diets from the same replicate population were paired in the CMH test.

To test whether allelic plasticity is overrepresented among genes showing abundance plasticity, we only considered genes that are included in both the allelic plasticity analysis and the abundance plasticity analysis. Significant abundance plasticity (i.e., differential expression between diets) was identified by *DESeq2* (*p* < 0.05 for “diet” effect on expression). We calculated the fraction of genes showing allelic plasticity amongst genes with significant abundance plasticity (*f*_1_) and the fraction of genes showing allelic plasticity amongst genes without significant abundance plasticity (*f*_2_). The difference between the two fractions is *diff_f* = *f*_1_ –*f*_2_.

To evaluate whether the observed *diff_f* is significantly different than expected by chance, we used permutations to produce an empirical null distribution of *diff_f* for each regime. For each site within each population, we permutated the two alleles across diets, keeping total read count within each diet unchanged. Each permuted site was tested by CMH test for allelic bias expression. “Significant” genes in this permutation test result from chance associations (i.e., classic false positives) but may be more likely to occur for some genes than others (i.e., genes with low coverage in one or both diets). The number of pseudo-significant genes was much lower than the observed number of sites with significant allelic plasticity. To complete the enrichment test, we designated “significant allelic bias” to randomly chosen sites until the total number of genes with “significant allelic bias” was the same as the actual number. The genes with significant abundance expression plasticity were based on the actual data (i.e., this feature of the data was not permuted). For each permuted data set, we calculate the difference in fraction of allelic expression bias genes between genes with or without significant abundance expression plasticity (*diff_f_permuted*). We performed 5000 permutations to generate the distribution of *diff_f_permuted* for each regime. The *p*-value was computed as the twice of the proportion of the permuted statistics that were equal or more extreme than the actual *diff_f*.

### Examining gene expression divergence and plasticity with respect to SNP differentiation

We obtained sites that are significantly differentiated with respect to genotypic SNP frequencies between cadmium- and salt-selected populations from previously reported genotypic sequencing data at generation 42 [20]. To determine whether the genes with ecologically differentiated SNPs are enriched for genes showing differential expression across regimes or diets, we only analyzed genes that not only have polymorphic SNPs for the allele frequency differentiation test [20] but are also involved in differential expression analysis. Further, we divided the genes into different categories based on whether the polymorphic SNPs are located in exons, introns and 1kb upstream and downstream of the genic region for enrichment test; genes with multiple SNP-types (e.g., SNPs in both exons and introns) are used in each relevant test. *χ*^2^ tests were used to test for significant enrichment.

In addition, we searched for evidence for adaptive differential expression of alternative alleles between diets. To obtain high statistical power, we identified SNPs that show significant genomic allele frequency differentiation between the six cadmium- and six salt-selected populations (by including *AC* and *AS*), only using the sites identified as having alternative alleles that are differentially expressed between diets, i.e., allelic plasticity (*p* < 0.01). For the set of genes that show both allelic plasticity and significant genomic allele frequency differentiation, we examined whether the direction of allelic plasticity between cadmium and salt diets is aligned with the direction of allele frequency change between cadmium- and salt-selected populations. For each of these genes, the allelic bias expression was assigned as positive if the direction of change between environments is the same, otherwise it was negative. The average allelic bias expression in the direction of allele frequency change was tested based on whether the bootstrapped distribution overlaps with 0.

## Supporting Information

S1 Information Gene-level pairwise comparisons among all four selective treatments. (DOCX)

S1 Fig **The log2 fold change (log**_**2**_**FC) between diets in *GA* vs. the log**_**2**_**FC between regimes *Cad* and *Salt* considering only the cadmium diet assay (A) and only the salt diet assay (B).** The log_2_FC between diets in *GA* vs. log_2_FC between diets considering only the *Cad* populations (C) and considering only the *Salt* populations (D).
(TIFF)

S2 Fig Cartoon example showing the potential inconsistency between adaptedness and plasticity for genes expected to evolve decreased plasticity. The optimal expressions (open symbols, *A*) are similar in two diets (*C* and *S*). Initially (left), expression is too high in each environment but differs between environments (i.e., plasticity exists). If the population experiences a heterogeneous environment, then in the long term, expression in each environment is expected evolve to a lower level and ultimately plasticity should disappear if optimal expression is reached in each environment. However, if the population proceeds faster in one diet (*C*) than the other (*S*), as shown in the right panel, then plasticity could be increased compared to the initial states. This may represent a transitory condition or a permanent one if it is not possible to reach the optimum in the *S* environment.
(TIFF)

S3 Fig The first and second principle components using all the samples, including ancestral populations. Different colors indicate samples from different regimes. Different shapes indicate samples from cadmium (circles) or salt (triangle) diet. The samples of the three ancestors, assayed together in a separate block, are somewhat distinct from those of the experimental populations.
(TIFF)

S4 Fig The first and second principle components for the PCA using only the experimental replicate samples. It shows the similarity within and among blocks. Different colors indicate samples collected at different blocks. This figure illustrates the lack of block effects (no clustering by color). Fig 3 depicts the results of the PCA with respect to selective regime.
(TIFF)

S1 TablePairwise differentiation between regimes with respect to mean expression across diets $\left(\bar{PC}\right)$ and plasticity between diets *(*Δ*PC)*.This table is intended to highlight the axes with the most differentiation for each regime pair based on visual inspection of the last two columns of Fig 3; it does not represent a formal statistical comparison.(DOCX)

S2 Table GO terms associated with high positive or negative loading values on each PC axis. In cases where more than 15 significant GO terms (FDR(*q*) < 0.05) were identified, only the 15 most significant GO terms are shown.
(DOCX)

S3 Table The number of genes with significant effects for each pair of selective regimes. The first number in each cell shows the genes with a significant “selective history” effect on expression. The number in square brackets shows the number of genes having significantly differentiated SNP frequencies between each pair (based on results from Huang et al. [20]). The second line shows the genes with a significant “diet” effect. The third line shows the genes with a significant “selective history × diet” interaction. The number in round brackets represents the number of genes with significant interaction effects where the diet effect goes in opposite directions for the two contrasted regimes. Here we use FDR < 0.1 to identify significant effects.)
(DOCX)

S4 Table The number of genes having a significant “selective history” effect (FDR < 0.1) for each pair of regime in the cadmium diet (top line) and the salt diet (bottom line). These data come from analyzing each assay diet separately.
(DOCX)

S5 Table GO terms associated with high expression divergence between regimes. For the pair of regimes (*Salt*-*Temp*) that have more than 15 significant GO terms (FDR(*q*) < 0.05), only the 15 most significant GO terms are shown.
(DOCX)

S6 Table GO terms associated with strongly differential expression between cadmium and salt diets for each regime. In cases where more than 15 significant GO terms (FDR(*q*) < 0.05) were identified, only the 15 most significant GO terms are shown.
(DOCX)

S7 Table Names for the genes used in comparing ancestral plasticity and evolved divergence (left), the genes predicted to evolve increased (middle) or decreased plasticity (right). (DOCX)
