# Supplementary material for: Experimental Evolution of Gene Expression and Plasticity in Alternative Selective Regimes
Source: PLoS Genet. 2016 Sep 23;12(9):e1006336. doi: 10.1371/journal.pgen.1006336 (PMC5035091; doi:10.1371/journal.pgen.1006336)
Supplement: S1 Information — (DOCX) [file pgen.1006336.s001.docx]

## Supplementary information

### Gene-level pairwise comparisons among all four selective treatments

To explore patterns of gene expression evolution and plasticity, we performed gene-level pairwise comparisons among all four selective regimes. For each gene, we examined a simple linear model: Expression ~ diet + selective history + diet × selective history + block. The number of genes with a significant “selective history” effect (FDR < 0.1) for each pair of regimes is shown in the first line in each cell in S2 Table. For comparison, the numbers of genes having significantly differentiated SNP frequencies from previous genomic results [20] are given in square brackets. Both expression data and allele frequency data suggest that *Salt* and *Cad* regimes are most differentiated amongst all pairings whereas the two heterogeneous regimes, *Temp* and *Spatial*, are the least differentiated. There is much higher expression divergence between *Salt* and each heterogeneous regime than between *Cad* and each heterogeneous regime, suggesting that the expression patterns of heterogeneous regimes are much closer to *Cad* than to *Salt* (consistent with the pattern in Fig 3). However, this asymmetry is not observed for the genomic divergence. If differential expression between regimes is analyzed separately for each diet, we see that most of the divergence between *Salt* and other regimes is evident only in cadmium (S3 Table), whereas *Cad* shows low divergence from other regimes in both diets. Significant GO terms for genes showing a “history” effect are shown in S5 Table.

The second line in each cell in S2 Table shows the number of genes with a significant diet effect. In contrast to selective history effects, the number of genes with a diet effect in the homogeneous pair (*Cad* vs. *Salt*) is similar to the number in the heterogeneous pair (*Temp* vs. *Spatial*). The pairs involving the *Salt* regime have the fewest genes showing a diet effect, which might due to *Salt* being affected differently by diets than the heterogeneous regimes (i.e., an interaction effect). The numbers of genes with a significant selective history × diet interaction are shown on the third line of each cell in S2 Table. Pairs involving the *Salt* regime tend to have the largest number of interaction effects, and many of these are cases where the effect of diet is in the opposite direction for the *Salt* regime compared to the other treatment in its pair. Significant GO terms for genes showing a strong “diet” effect for each regime are shown in S6 Table.
