# Supplementary figures and images for: Experimental Evolution of Gene Expression and Plasticity in Alternative Selective Regimes
Source: PLoS Genet. 2016 Sep 23;12(9):e1006336. doi: 10.1371/journal.pgen.1006336 (PMC5035091; doi:10.1371/journal.pgen.1006336)

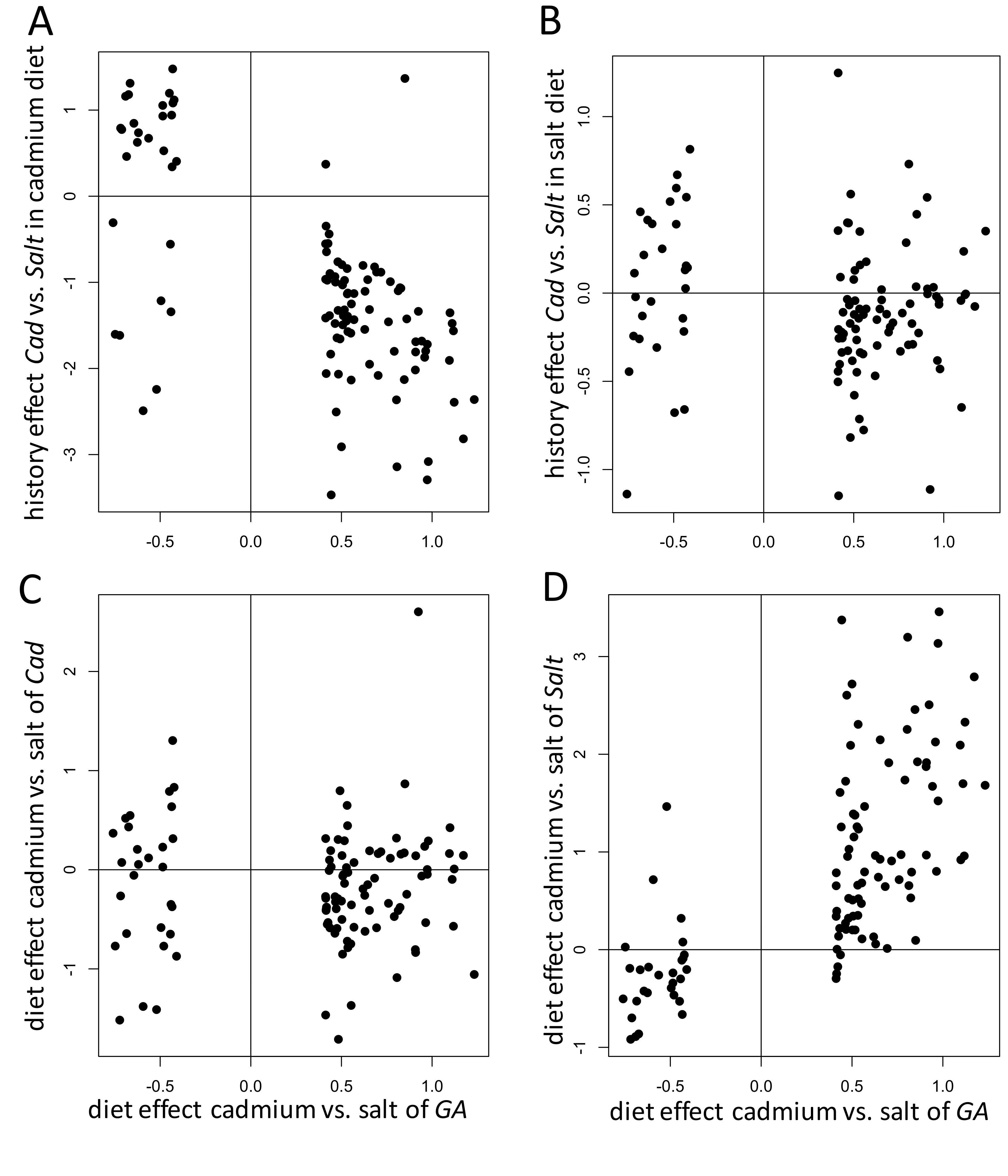

Supplement: S1 Fig — The log2 fold change (log2FC) between diets in GA vs. the log2FC between regimes Cad and Salt considering only the cadmium diet assay (A) and only the salt diet assay (B). The log2FC between diets in GA vs. log2FC between diets considering only the Cad populations (C) and considering only the Salt populations (D). (TIFF) [file pgen.1006336.s002.tiff]

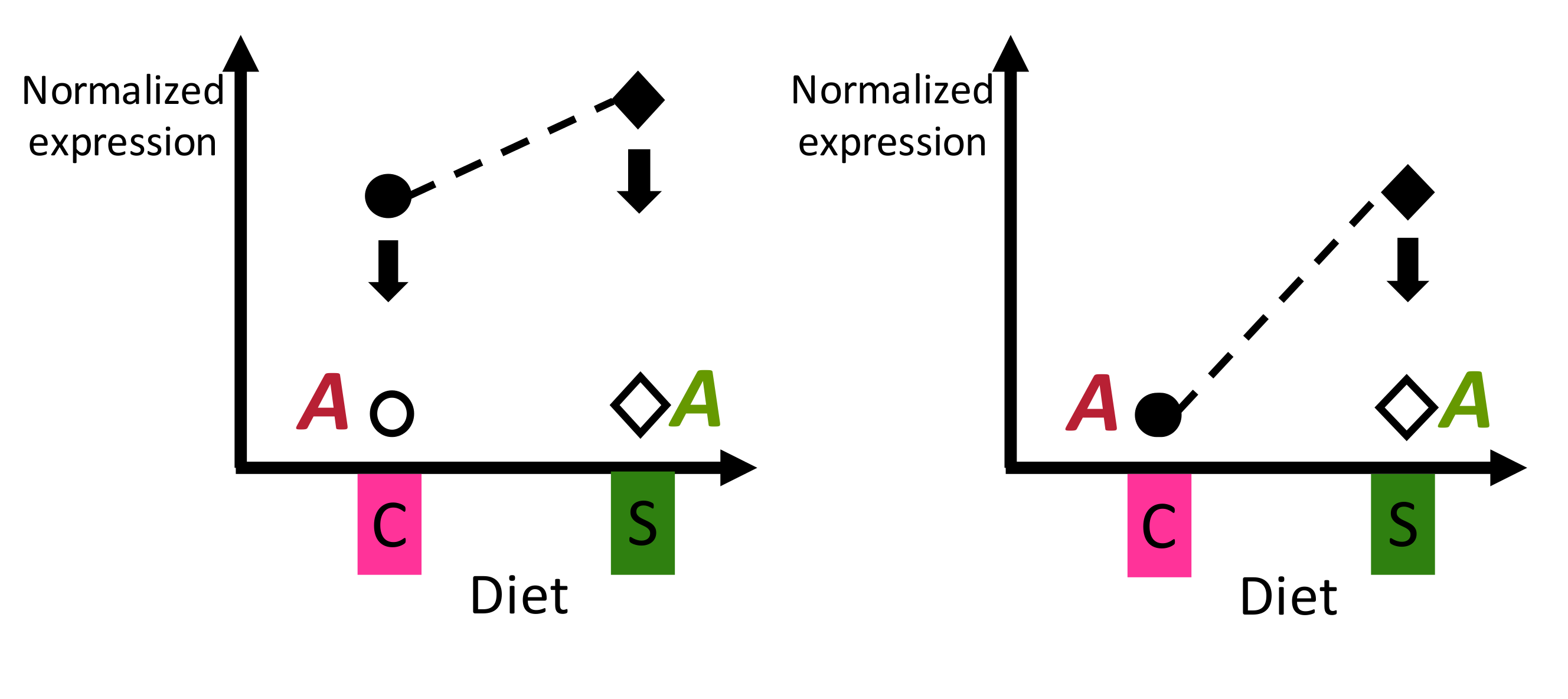

Supplement: S2 Fig — The optimal expressions (open symbols, A) are similar in two diets (C and S). Initially (left), expression is too high in each environment but differs between environments (i.e., plasticity exists). If the population experiences a heterogeneous environment, then in the long term, expression in each environment is expected evolve to a lower level and ultimately plasticity should disappear if optimal expression is reached in each environment. However, if the population proceeds faster in one diet (C) than the other (S), as shown in the right panel, then plasticity could be increased compared to the initial states. This may represent a transitory condition or a permanent one if it is not possible to reach the optimum in the S environment. (TIFF) [file pgen.1006336.s003.tiff]

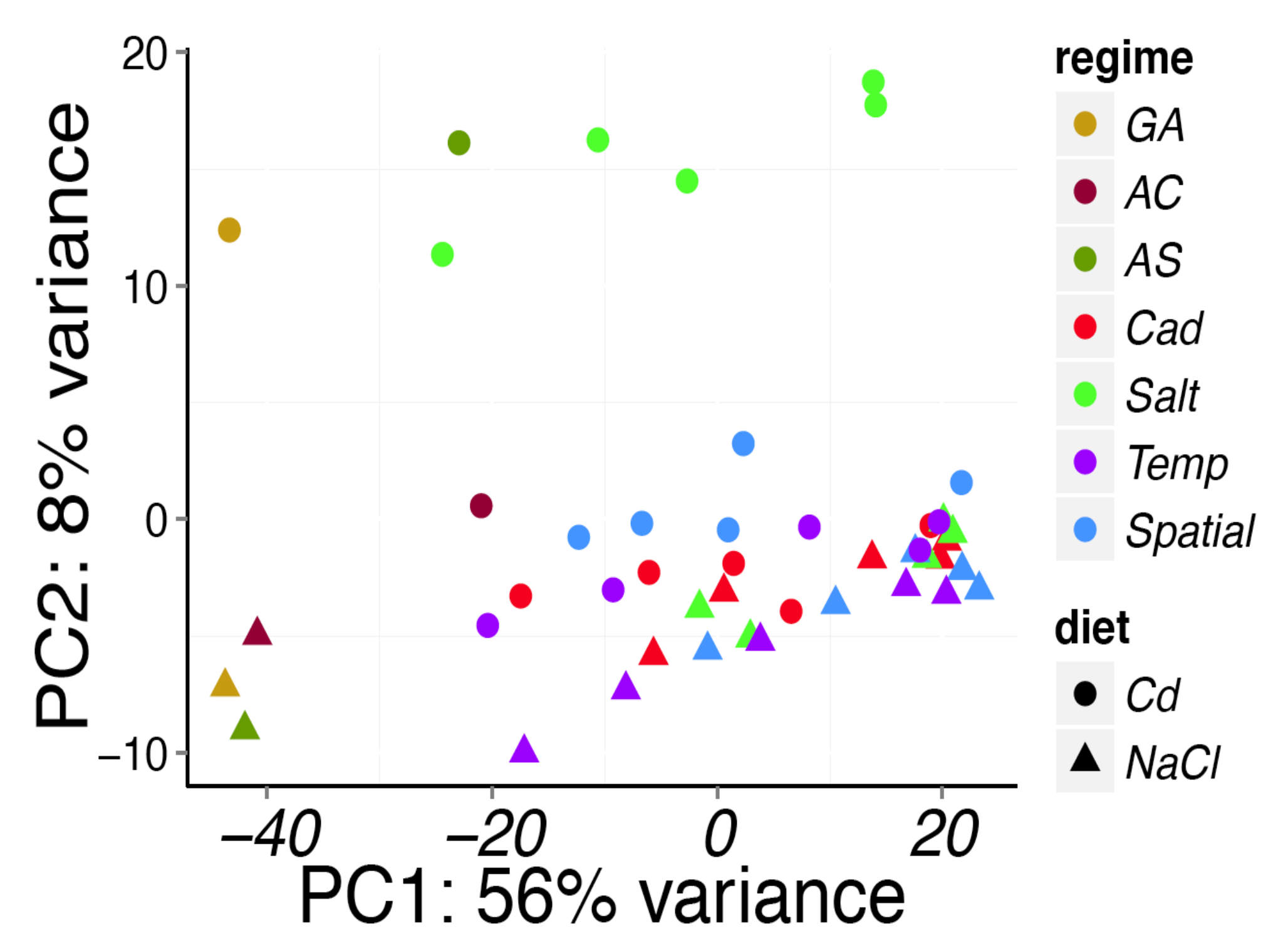

Supplement: S3 Fig — Different colors indicate samples from different regimes. Different shapes indicate samples from cadmium (circles) or salt (triangle) diet. The samples of the three ancestors, assayed together in a separate block, are somewhat distinct from those of the experimental populations. (TIFF) [file pgen.1006336.s004.tiff]

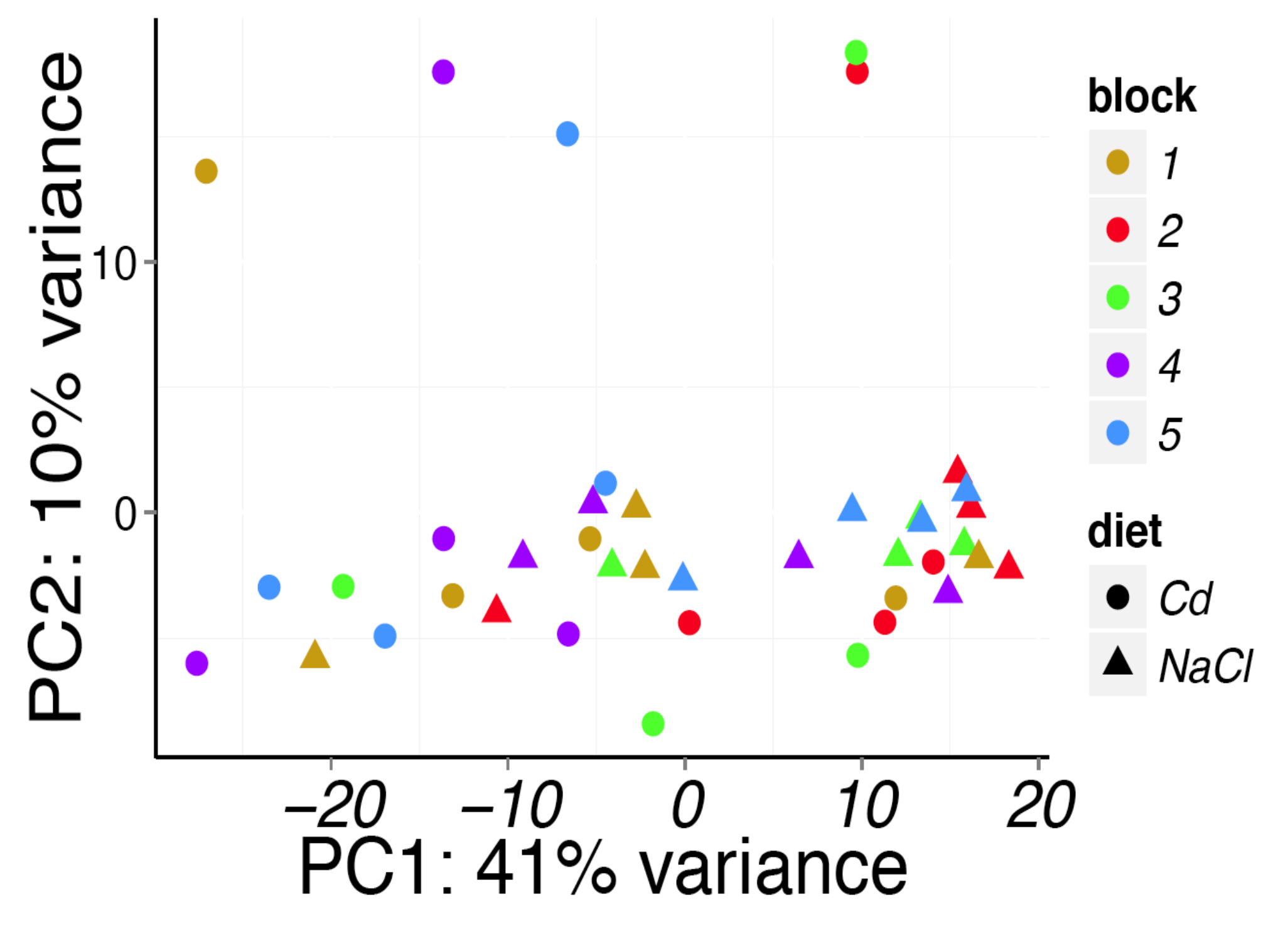

Supplement: S4 Fig — It shows the similarity within and among blocks. Different colors indicate samples collected at different blocks. This figure illustrates the lack of block effects (no clustering by color). Fig 3 depicts the results of the PCA with respect to selective regime. (TIFF) [file pgen.1006336.s005.tiff]
