# Supplementary material for: Experimental Evolution of Gene Expression and Plasticity in Alternative Selective Regimes
Source: PLoS Genet. 2016 Sep 23;12(9):e1006336. doi: 10.1371/journal.pgen.1006336 (PMC5035091; doi:10.1371/journal.pgen.1006336)
Supplement: S1 Table — This table is intended to highlight the axes with the most differentiation for each regime pair based on visual inspection of the last two columns of Fig 3; it does not represent a formal statistical comparison. (DOCX) [file pgen.1006336.s006.docx]

**Supplementary Tables 1**

|  | ***Cad*** | ***Temp*** | ***Spatial*** |
| --- | --- | --- | --- |
| ***Salt*** | $\bar{PC2}, \Delta PC2$ | $\bar{PC2}, \Delta PC2$ | $\bar{PC2}, \Delta PC2$ |
| ***Cad*** |  | $\bar{PC4}$ | $\Delta PC2, \Delta PC5$ |
| ***Temp*** |  |  | $\bar{PC2}, \bar{PC4},$ |
