# Supplementary material for: Experimental Evolution of Gene Expression and Plasticity in Alternative Selective Regimes
Source: PLoS Genet. 2016 Sep 23;12(9):e1006336. doi: 10.1371/journal.pgen.1006336 (PMC5035091; doi:10.1371/journal.pgen.1006336)
Supplement: S2 Table — In cases where more than 15 significant GO terms (FDR(q) < 0.05) were identified, only the 15 most significant GO terms are shown. (DOCX) [file pgen.1006336.s007.docx]

Supplementary Table 2

| Enriched GO term (*q* < 0.05) | Positive loading | Negative loading |
| --- | --- | --- |
| PC1 | [1] "integral component of membrane"  [2] “membrane”  [3] "molecular_function"  [4] "integral component of plasma membrane"  [5] "biological_process" | [1] "structural constituent of ribosome" [2] "translation"  [3] "ribosome" [4] "cytosolic large ribosomal subunit"  [5] "cytosolic small ribosomal subunit" [6] "mitotic spindle organization"  [7] "mitotic spindle elongation" [8] "centrosome duplication"  [9] "microtubule associated complex" [10] "lipid particle"  [11]"translational elongation" [12] "proton transport"  [13]"mitochondrion" [14] "hydrogen-exporting ATPase activity, phosphorylative mechanism"  [15] "translation elongation factor activity" |
| PC2 | [1]"glutathione transferase activity" | [1] "structural constituent of ribosome" [2] "mitochondrion"  [3]"translation" [4] "microtubule associated complex"  [5] "mitotic spindle organization" [6] "lipid particle"  [7]"protein folding" [8] "nucleolus"  [9]"ribosome" [10]"peptidyl-prolyl cis-trans isomerase activity"  [11]"ribonucleoprotein complex" [12] "mitotic spindle elongation"  [13] "hydrogen-exporting ATPase activity, phosphorylative mechanism" [14] "centrosome duplication"  [15] "nucleus" |
| PC3 | [1] "nucleus"  [2] "protein folding" | [1] "structural constituent of ribosome" [2] "ribosome" [3] "translation"  [4] "cytosolic small ribosomal subunit" [5] "cytosolic large ribosomal subunit" |
| PC4 | NA | [1] "carboxylic ester hydrolase activity" |
| PC5 | NA | NA |
