# Supplementary material for: Experimental Evolution of Gene Expression and Plasticity in Alternative Selective Regimes
Source: PLoS Genet. 2016 Sep 23;12(9):e1006336. doi: 10.1371/journal.pgen.1006336 (PMC5035091; doi:10.1371/journal.pgen.1006336)
Supplement: S3 Table — The first number in each cell shows the genes with a significant “selective history” effect on expression. The number in square brackets shows the number of genes having significantly differentiated SNP frequencies between each pair (based on results from Huang et al. [20]). The second line shows the genes with a significant “diet” effect. The third line shows the genes with a significant “selective history × diet” interaction. The number in round brackets represents the number of genes with significant interaction effects where the diet effect goes in opposite directions for the two contrasted regimes. Here we use FDR < 0.1 to identify significant effects.) (DOCX) [file pgen.1006336.s008.docx]

Supplementary Table 3

| **Regime** | ***Cad*** | ***Temp*** | ***Spatial*** |
| --- | --- | --- | --- |
| ***Salt*** | 546 [5560]  724  563 (487) | 431 [1471]  291  356 (316) | 322 [1305]  692  161 (135) |
| ***Cad*** |  | 68 [1779]  919  2 (2) | 56 [802]  1205  4 (2) |
| ***Temp*** |  |  | 32 [180]  678  0 |
