# Supplementary material for: Experimental Evolution of Gene Expression and Plasticity in Alternative Selective Regimes
Source: PLoS Genet. 2016 Sep 23;12(9):e1006336. doi: 10.1371/journal.pgen.1006336 (PMC5035091; doi:10.1371/journal.pgen.1006336)
Supplement: S4 Table — These data come from analyzing each assay diet separately. (DOCX) [file pgen.1006336.s009.docx]

Supplementary Table 4

| **Regime** | ***Cad*** | ***Temp*** | ***Spatial*** |
| --- | --- | --- | --- |
| ***Salt*** | 588  40 | 477  3 | 248  5 |
| ***Cad*** |  | 16  13 | 6  10 |
| ***Temp*** |  |  | 4  9 |
