# Supplementary material for: Experimental Evolution of Gene Expression and Plasticity in Alternative Selective Regimes
Source: PLoS Genet. 2016 Sep 23;12(9):e1006336. doi: 10.1371/journal.pgen.1006336 (PMC5035091; doi:10.1371/journal.pgen.1006336)
Supplement: S5 Table — For the pair of regimes (Salt-Temp) that have more than 15 significant GO terms (FDR(q) < 0.05), only the 15 most significant GO terms are shown. (DOCX) [file pgen.1006336.s010.docx]

Supplementary Table 5

| Expression divergence | Up-regulated in first regime | Up-regulated in second regime |
| --- | --- | --- |
| *Cad-Salt* | [1] "structural constituent of ribosome"  [2] "mRNA splicing, via spliceosome"  [3] "mRNA binding"  [4] "proteasome regulatory particle" | None |
| *Cad-Temp* | None | [1] "structural constituent of ribosome"  [2] "ribosome"  [3] "translation"  [4] "cytosolic large ribosomal subunit"  [5] "cytosolic small ribosomal subunit"  [6] "mitotic spindle elongation"  [7] "lipid particle" |
| *Cad-Spatial* | [1] "ribosome" [2] "structural constituent of ribosome" | None |
| *Salt-Temp* | None | [1] "structural constituent of ribosome"  [2] "translation"  [3] "ribosome" [4] "cytosolic large ribosomal subunit"  [5] "mitotic spindle elongation"  [6] "microtubule associated complex"  [7] "lipid particle" [8] "mitotic spindle organization"  [9] "mRNA binding" [10] "translation initiation factor activity"  [11] "cytosolic small ribosomal subunit" [12] "mRNA splicing, via spliceosome"  [13] "mitochondrion" [14] "nucleolus"  [15] "endopeptidase activity" |
| *Salt-Spatial* | [1] "glutathione transferase activity"  [2] "neuropeptide signaling pathway" | [1] "COP9 signalosome"  [2] "lysozyme activity" |
| *Temp-Spatial* | [1] "structural constituent of ribosome"  [2] "ribosome" [3] "translation"  [4] "cytosolic large ribosomal subunit"  [5] "mitotic spindle elongation"  [6] "lipid particle"  [7] "cytosolic small ribosomal subunit"  [8] "microtubule associated complex"  [9] "mitotic spindle organization" | None |
