# Supplementary material for: Experimental Evolution of Gene Expression and Plasticity in Alternative Selective Regimes
Source: PLoS Genet. 2016 Sep 23;12(9):e1006336. doi: 10.1371/journal.pgen.1006336 (PMC5035091; doi:10.1371/journal.pgen.1006336)
Supplement: S6 Table — In cases where more than 15 significant GO terms (FDR(q) < 0.05) were identified, only the 15 most significant GO terms are shown. (DOCX) [file pgen.1006336.s011.docx]

Supplementary Table 6

| Regime | Up-regulated in cadmium diet | Up-regulated in salt diet |
| --- | --- | --- |
| *Cad* | [1] "structural constituent of ribosome"  [2] "translation"  [3] "ribosome"  [4] "cytosolic large ribosomal subunit"  [5] "cytosolic small ribosomal subunit"  [6] "mitotic spindle elongation"  [7] "microtubule associated complex"  [8] "mitotic spindle organization"  [9] "centrosome duplication"  [10] "lipid particle"  [11] "mRNA binding"  [12] "translation initiation factor activity"  [13] "eukaryotic translation initiation factor 3 complex"  [14] "ribonucleoprotein complex"  [15] "hydrogen-exporting ATPase activity, phosphorylative mechanism" | [1] "electron carrier activity"  [2] "oxidoreductase activity, acting on paired donors, with incorporation or reduction of molecular oxygen"  [3] "heme binding"  [4] "ecdysteroid metabolic process"  [5] "oxidoreductase activity"  [6] "oxidation-reduction process" |
| *Salt* | [1] "ribosome"  [2] "structural constituent of ribosome"  [3] "cytosolic large ribosomal subunit"  [4] "cytosolic small ribosomal subunit"  [5] "translation"  [6] "mitotic spindle elongation"  [7] "glutathione transferase activity"  [8] "lipid particle"  [9] "imaginal disc-derived wing morphogenesis"  [10] "actin binding"  [11] "centrosome duplication"  [12] "translational elongation"  [13] "basement membrane"  [14] "axon guidance"  [15] "sarcomere organization" | None |
| *Temp* | [1] "protein folding"  [2] "nucleolus"  [3] "endopeptidase activity"  [4] "mRNA splicing, via spliceosome"  [5] "DNA-dependent DNA replication"  [6] "structural constituent of ribosome" | [1] "ecdysteroid metabolic process" |
| *Spatial* | [1] "structural constituent of ribosome"  [2] "translation"  [3] "ribosome"  [4] "cytosolic large ribosomal subunit"  [5] "mitotic spindle elongation"  [6] "cytosolic small ribosomal subunit"  [7] "lipid particle"  [8] "mRNA binding"  [9] "microtubule associated complex"  [10] "mitotic spindle organization"  [11] "translation initiation factor activity"  [12] "centrosome duplication"  [13] "endopeptidase activity"  [14] "translational initiation"  [15] "nucleolus" | None |
