# Supplementary material for: Experimental Evolution of Gene Expression and Plasticity in Alternative Selective Regimes
Source: PLoS Genet. 2016 Sep 23;12(9):e1006336. doi: 10.1371/journal.pgen.1006336 (PMC5035091; doi:10.1371/journal.pgen.1006336)
Supplement: S7 Table — (DOCX) [file pgen.1006336.s012.docx]

Supplementary Table 7

| Genes involved in comparing the ancestral plasticity and evolved divergence | Genes expected to evolve increased plasticity | Genes expected to evolve decreased plasticity |
| --- | --- | --- |
| *E2f2*  *Gr39a*  *CG10337*  *CG8773*  *rab3-GEF*  *sina*  *CG10237*  *rha*  *Calx*  *tadr*  *CG15523*  *Alas*  *CG13202*  *Npc2e*  *CG5112*  *CG10069*  *CG9855*  *CG18445*  *CG6996*  *Ciao1*  *CG14372*  *FucTC*  *sinah*  *CG31793*  *lectin-46Ca*  *CG33483*  *nAcRbeta-21C*  *CG3500*  *Nlp*  *CG18173*  *Hsromega*  *CG14645*  *CG3520*  *Npc2b*  *CG3608*  *lectin-46Cb*  *SmD3*  *CG11023*  *CG13248*  *CG31202*  *Trx-2*  *CG6499*  *Magi*  *CG1774*  *LIMK1*  *Tsf2*  *CG31810*  *esn*  *CG31549*  *CG5002*  *hiw*  *CG13404*  *PEK*  *CG11852*  *CG17985*  *CG14044*  *CG10132*  *CG8944*  *Fer2LCH*  *CG9265*  *CG42239*  *CG9821*  *CR31514*  *CG42588*  *Kif3C*  *CG4301*  *Arp66B*  *CG4210*  *CG10898*  *Pis*  *CG7039*  *Ost48*  *CG9454*  *CG13397*  *CG7857*  *pst*  *CG1208*  *Gycalpha99B*  *nop5*  *dome*  *CG10880*  *CG34263*  *alien*  *CG6484*  *CG3987*  *CG4433*  *eIF2B-gamma*  *rtet*  *CG12182*  *CG13676*  *CG13510*  *VhaM9.7-2*  *dos*  *CG17660*  *Syn*  *CG18095*  *Mctp*  *CG17119*  *betaggt-I*  *CG30381*  *CG12963*  *CG2129*  *yellow-g*  *Csp*  *CG7685*  *CG1671*  *CG14906*  *CalpB* | *CG31360*  *CG15449*  *CG10927*  *CG31304*  *Cyp4s3*  *CG13313*  *CG11771*  *DopEcR*  *CG17209*  *CG14372*  *CG6904*  *Pig1*  *CG18223*  *CG5541*  *CG33998*  *CG5734*  *CG10903*  *CG5337*  *abd-A*  *CG42525*  *CG11784*  *CG34431*  *CG32428*  *CG31643*  *CG42330*  *brp*  *elgi*  *tadr*  *CG3074*  *CG16868*  *TFAM*  *CG9945*  *Vap-33-1*  *CG8671*  *MED1*  *CG42568*  *CG10598*  *CG3288*  *CG31156*  *Cpr67Fb*  *Ptp52F*  *Cyp4d2*  *spheroide*  *psidin*  *CG13397*  *CG2790*  *how*  *Trim9*  *CadN2*  *mRpL23*  *CG6357*  *Dak1*  *CycT*  *CG34282*  *Tom34*  *CG13921*  *Mst77F*  *CG5009*  *mRpS29*  *unc-13*  *CG5381*  *CG11437*  *dsh*  *CG17104*  *CG7408*  *mnd*  *CG2135*  *Cyp12a4*  *CG4038*  *CG12643*  *CG1172*  *Or13a*  *Pc*  *Cyp49a1*  *Scm*  *CG14195*  *Hex-C*  *CG15528*  *CG32647*  *CG13843*  *CG9306*  *Pka-R2*  *Top3beta*  *CG15073*  *CG34408*  *dac*  *Ir21a*  *sbr*  *CG5780*  *Gyc-89Da*  *CG14872*  *CG14285*  *Bsg25D*  *Cyp4ae1*  *Sb*  *inaF-C*  *tkv*  *CG18324*  *nocturnin*  *Sry-delta*  *Rassf*  *ninaC*  *CG14411*  *CG5220*  *CG7910*  *CG10672*  *Prat*  *CG7394*  *CG32409* | *CG9705*  *CG9313*  *Tap42*  *CG32369*  *CG2931*  *CG1319*  *alpha-Man-II*  *CG17119*  *CG32789*  *l(2)35Cc*  *CG15082*  *Mdr50*  *Or85f*  *Nlp*  *CG5104*  *Myo31DF*  *CG42362*  *Tsp42Ed*  *CG9279*  *CG8993*  *tilB*  *CG8329*  *ALiX*  *JIL-1*  *CG3744*  *CG11357*  *boca*  *RpI12*  *TwdlG*  *CG17221*  *CG1635*  *obst-A*  *CG17598*  *dnc*  *alpha-Est1*  *Gmer*  *pip*  *CG31104*  *CG7791*  *slv*  *CG4294*  *InR*  *Hr96*  *Sans*  *CG18522*  *CG18317*  *beta4GalNAcTA*  *CG9380*  *DNApol-iota*  *CG32662*  *mRpS33*  *Elf*  *Pak3*  *CaBP1*  *CG31075*  *CycD*  *CG8359*  *CG33181*  *CG17768*  *CG6765*  *CG14939*  *Elo68alpha*  *Rpd3*  *RN-tre*  *CG30105*  *mRpL30*  *CG14104*  *CG3781*  *Hsp26*  *Tango10*  *CG17974*  *CG10182*  *Ilp6*  *CG1638*  *CG11658*  *mof*  *CG30503*  *CG11168*  *CG9836*  *CG31033*  *CG42379*  *CG8907*  *CG10625*  *twin*  *Trf4-1*  *CG5190*  *Unr*  *Arc1*  *Neu3*  *4-Sep*  *CG5267*  *UbcD6*  *Gclc*  *CG10669*  *Pal*  *ImpL2*  *Hsp70Aa*  *Df31*  *Cyp9b2*  *CG6028*  *Cad96Cb*  *Rpb4*  *CG16998*  *CG3825*  *Ide*  *CG4839*  *CG1824*  *CG14273*  *CG8788*  *CG1662*  *CG14439*  *CG4853*  *kat-60L1*  *FK506-bp1*  *CG11637*  *CG17282*  *GluClalpha*  *CG12111*  *CG11722*  *mri*  *CG17109* |
